# High Performance, Fully Bio‐Based, and Optically Transparent Wood Biocomposites

**DOI:** 10.1002/advs.202100559

**Published:** 2021-05-02

**Authors:** Céline Montanari, Yu Ogawa, Peter Olsén, Lars A. Berglund

**Affiliations:** ^1^ Department of Fibre and Polymer Technology Wallenberg Wood Science Center KTH Royal Institute of Technology Teknikringen 56 Stockholm 10044 Sweden; ^2^ Université Grenoble Alpes CNRS CERMAV Grenoble 38000 France

**Keywords:** bio‐based polymers, biocomposite, nanotechnology, sustainable, transparent wood

## Abstract

The sustainable development of engineering biocomposites has been limited due to a lack of bio‐based monomers combining favorable processing with high performance. Here, the authors report a novel and fully bio‐based transparent wood biocomposite based on green synthesis of a new limonene acrylate monomer from renewable resources. The monomer is impregnated and readily polymerized in a delignified, succinylated wood substrate to form optically transparent biocomposites. The chemical structure of the limonene acrylate enables diffusion into the cell wall, and the polymer phase is both refractive index‐matched and covalently linked to the wood substrate. This results in nanostructured biocomposites combining an excellent optical transmittance of 90% at 1.2 mm thickness and a remarkably low haze of 30%, with a high mechanical performance (strength 174 MPa, Young's modulus 17 GPa). Bio‐based transparent wood holds great potential towards the development of sustainable wood nanotechnologies for structural applications, where transparency and mechanical performance are combined.

## Graphical abstract

A fully bio‐based transparent wood biocomposite is prepared by impregnation and polymerization of a novel, green limonene acrylate monomer into a wood substrate. Succinylation of the delignified wood substrate using succinic anhydride results in a nanostructured and mechanically strong biocomposite with a tailored interface. The optically transparent wood biocomposite combines high transmittance and low haze.

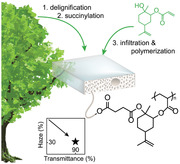

## 1 Introduction

The development of green materials from renewable resources, combining strength with functional properties, is essential for sustainable development. Wood is an attractive starting point because of its high mechanical performance and it is lightweight, stemming from its porous and anisotropic cellulosic structure; and is of great interest for large‐scale sustainable nanotechnologies.^[^ ^1^ ^]^ Engineered transparent wood is an example of recent wood nanotechnology for advanced biocomposite applications.^[^ ^2^, ^3^, ^4^ ^]^ Recent studies of transparent wood (TW) have revealed attractive anisotropic optical properties,^[^ ^5^, ^6^, ^7^ ^]^ with optical transmittance above 90% at 1.2 mm thickness^[^ ^8^ ^]^ and high haze (80%) provision of diffused light,^[^ ^9^ ^]^ lower thermal conductivity than glass,^[^ ^10^ ^]^ and excellent mechanical strength (270 MPa) at a wood volume fraction of ≈30 vol%.^[^ ^11^ ^]^ Multifunctional TW composites, often in photonics applications, can be developed by the incorporation of nanoparticles,^[^ ^12^, ^13^, ^14^ ^]^ quantum dots,^[^ ^15^ ^]^ lasing dyes,^[^ ^16^ ^]^ phase‐change materials,^[^ ^17^ ^]^ or conductive polymers.^[^ ^18^ ^]^

The general preparation approach for TW biocomposites consists of first removing the light‐absorbing chromophores from the wood substrate, followed by infiltration of a petrochemical polymer precursor, forming a polymer with refractive index close to that of wood, such as PMMA or commercial epoxies.^[^ ^5^, ^9^ ^]^ While renewable reinforcement like wood reduces the environmental impact of composites, the overall sustainability of wood biocomposites can still be improved because of the fossil‐based polymer matrix. The polymer matrix usually accounts for ≈70 vol%, even for transparent wood composites based on high‐density wood species such as birch. Therefore, to avoid petroleum resources and reduce carbon footprint, bio‐based polymer matrices are desirable.^[^ ^19^ ^]^ Note that the biodegradability aspect of the polymer matrix is not a concern here, because of durability requirements and long service life of most transparent wood applications considered, for example, in buildings.

Bio‐based polymers can offer advantages compared to conventional non‐renewable polymers from fossil resources, including the use of renewable resources, carbon neutrality, and low global warming impact.^[^ ^20^ ^]^ In this context, terpenes are interesting for the synthesis of sustainable and bio‐based polymers.^[^ ^21^, ^22^, ^23^, ^24^, ^25^, ^26^, ^27^, ^28^ ^]^ Limonene is a common cyclic terpene that can be extracted from industrial waste, via isomerization of *α*‐pinene (from wood) or from citrus peel oil.^[^ ^29^, ^30^ ^]^ The recovery potential from citrus peel waste is estimated to 65 million metric tons per year.^[^ ^31^, ^32^ ^]^ Limonene‐based monomers have been reported for designing new bio‐based polymers, such as polycarbonates, polyesters, polyacrylates, polyamides, and polyurethanes.^[^ ^26^, ^27^, ^33^, ^34^, ^35^, ^36^, ^37^, ^38^, ^39^, ^40^ ^]^ Very few of these monomers are suitable for scalable in‐situ polymerization processing of composites, and toxic reactants or catalysts are often used. A green chemistry approach for bio‐based monomers is therefore highly desirable, where the polymerization strategy is tailored for cellulosic biocomposites.

Here, we report a green synthesis of a novel limonene acrylate (LIMA) monomer for the preparation of fully bio‐based transparent wood; where challenges related to polymerization side‐reactions, optical defects, and sufficiently high service temperature are addressed. The transparent and refractive‐index‐matched poly(limonene acrylate) (PLIMA) is impregnated into delignified wood substrates resulting in mechanically strong and highly transparent biocomposites. The mechanical and optical properties are further improved by tailoring the nanoscale interface between cellulose fibrils in the wood cell wall and PLIMA via green succinylation of the wood cell wall using bio‐based succinic anhydride. Succinylation facilitates molecular wood‐PLIMA interaction inside the wood cell wall, by allowing nanoscale LIMA impregnation, and covalently linking PLIMA to the modified wood substrate. The present objective is to achieve nanostructural control in a bio‐based wood composite system. The final biocomposites from different wood species are fully bio‐based transparent wood materials, not only with high optical transmittance, but also with the lowest haze reported for 1.2 mm thick specimens, and mechanical properties approaching those of petroleum‐based transparent wood composites. The universality of the approach is attractive for the development of sustainable building materials for load‐bearing applications; where the nanocomposite nature of the wood/PLIMA cell wall contributes favorably to high optical and mechanical performance.

## 2 Results and Discussion

### 2.1 Bio‐Based Limonene Acrylate Monomer

In transparent wood (TW) biocomposites, the reinforcing phase consists of a low‐density, anisotropic, porous wood substrate, which is filled by a polymeric “matrix”. For high performance applications, besides high wood volume fraction combined with optical transmittance, favorable load transfer between the matrix and wood reinforcement is required. Ideally, the polymer phase should be exclusively based on renewable resources; have a scalable and sustainable synthesis; show favorable molecular interactions with the wood substrate; be amorphous and optically transparent with a glass transition temperature *T*_g_ > 100°C for structural applications. From this perspective, acrylic monomers are suitable candidates as they fulfill most of these criteria. Another important advantage is that free radical polymerization of acrylics is suitable for scalable composites processing, and, unlike many others, insensitive to residual moisture in the wood substrate. Thus, our approach is to explore a completely new sustainable synthetic pathway towards a bulky acrylic monomer, based on the combination of acrylic acid and limonene oxide from renewable resources. The bio‐based monomer is designed to have both facile free radical polymerization behavior with late‐stage cross‐linking, and sufficient polarity to enter the cell wall in the wood substrate to improve compatibility.

**Figure 1:**
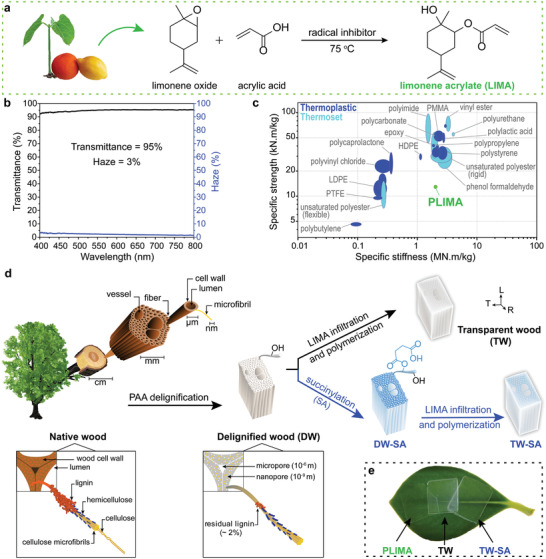
a) Chemical route for the synthesis of limonene acrylate (LIMA) monomer. b) Optical transmittance and haze of PLIMA. c) Mechanical properties of PLIMA bio‐based polymer compared with common thermosets. d) Schematic illustration showing the structure of wood and the various preparation steps of the bio‐based transparent wood (TW) and TW‐SA. First, a green peracetic acid (PAA) delignification is performed to remove the lignin, followed by succinylation for improved LIMA impregnation, using bio‐based succinic anhydride (SA) under neat conditions. Finally, LIMA monomer is infiltrated in the wood substrate and polymerized. e) Photograph of PLIMA, bio‐based TW, and TW‐SA.

We developed a new bio‐based limonene acrylate (LIMA) monomer that carries three different functionalities: an activated alkene to provide facile propagation kinetics, a deactivated second alkene to provide late‐stage cross‐linking, and a *β*‐hydroxyl to enable both diffusion into the substrate cell wall and chemical reaction with SA carboxyls in succinylated wood. **Figure** **1**a shows the synthesis pathway of the LIMA monomer, based on limonene oxide and acrylic acid. LIMA is synthesized via ring‐opening acrylation of limonene oxide, rather than the common route for acrylics using acyl chlorides. Acrylic acid can be produced from renewable resources, either by oxidation of acrolein, which is derived from glycerol, or through direct dehydration of lactic acid and 3‐hydroxypropionic acid.^[^ ^41^, ^42^ ^]^ Limonene oxide is simply derived by the oxidation of limonene. The ring‐opening acrylation of limonene oxide was performed under neat conditions for 3 h at 75 °C. LIMA was isolated at a yield above 85% after simple washing with Na_2_CO_3_. During reaction optimization, it was found that addition of 1 mol% of 4‐methoxyphenol suppressed any self‐polymerization. The ^1^H and ^13^C NMR spectra of the obtained monomer are detailed in Figure S1, Supporting Information. Overall, this synthetic pathway provides a scalable and sustainable route towards a new acrylic monomer, LIMA, based only on renewable resources.

LIMA was polymerized through free radical polymerization with 2,2′‐azobis(2‐methylpropionitrile) (AIBN) as the radical initiator. Importantly, this mechanism is insensitive to residual moisture in the wood substrate. The deactivated second alkene in the LIMA monomeric structure results in late‐stage chemical cross‐linking during polymerization, which leads to the formation of a 3D network and increased *T*_g_ (Scheme S1, Supporting Information). The resulting bio‐based PLIMA thermoset has a *T*_g_ of 131 °C, as determined by dynamic mechanical thermal analysis (DMTA) (see Figure S2, Supporting Information). High *T*_g_ is favorable for composite applications at elevated temperature. Other demanding criteria for a bio‐based polymer matrix in TW biocomposites are high optical transmittance and refractive index matching with the wood substrate (1.54).^[^ ^43^ ^]^ PLIMA has a suitable refractive index of 1.52 and demonstrates a high total transmittance of 95% combined with very low haze (forward‐scattered light) of 3% at a wavelength of 550 nm (Figure 1b). The optical properties are comparable with those of petroleum‐based transparent polymers for optics applications (Figure S3, Supporting Information). The Young's modulus is 2 GPa, which is reasonable for an amorphous acrylic resin, and makes PLIMA a bio‐based alternative to comparable petroleum‐based polymers (Figure 1c). PLIMA, like many highly cross‐linked thermosets, is brittle in uniaxial tension, where strain to failure and tensile strength is controlled by defects in the specimen,^[^ ^44^ ^]^ for instance edge flaws from the specimen cutting procedure.

### 2.2 Interface Tailoring and Preparation of Bio‐Based Transparent Wood

Bio‐based TW were prepared from hardwood species of various densities, including balsa (*Ochroma pyramidale)*, alder (*Alnus glutinosa*), birch (*Betula pendula*), and beech (*Fagus sylvestris*) wood. The wood structure is the reinforcement phase in TW, and the species were selected to vary wood volume fractions (*V*_f_), see **Table** **1**. The bio‐based TWs are fabricated by impregnating delignified wood (DW) and succinylated wood (DW‐SA) substrates by liquid LIMA monomer and AIBN radical initiator, followed by heating to initiate polymerization. Figure 1d illustrates fabrication routes to obtain two materials: the TW (without succinylation) and TW‐SA (with succinylation) biocomposites. Figure 1e shows the excellent optical transparency of the designed bio‐based PLIMA polymer and the prepared bio‐based TW and TW‐SA.

**Table 1:** Characteristics of the native wood (NW) and delignified wood (DW) substrates from various wood species, used as reinforcements in the transparent wood (TW) and TW‐SA biocomposites (TW without and TW‐SA with succinylation)

	Balsa	Alder	Birch	Beech
NW density [kg m^−3^]	304	510	593	631
DW density [kg m^−3^]	184	295	384	414
Weight loss after delignification [%]	34	32	26	26
DW *S*_BET_ [m^2^ g^−1^]	259	227	167	148
TW density [kg m^−3^]	1165	1232	1229	1233
TW‐SA density [kg m^−3^]	1182	1235	1240	1270
Wood volume fraction [%]	12	22	26	29

**Figure 2:**
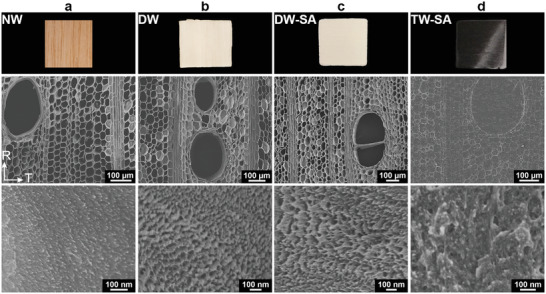
Photographs and SEM images of balsa showing a) native wood (NW), b) delignified wood (DW), c) succinylated‐delignified wood (DW‐SA), and d) succinylated transparent wood (TW‐SA) with the bio‐based PLIMA polymer as a matrix, where the high‐resolution cross‐sectional SEM micrographs at the bottom show the interior of the cell wall.

In Figure 1d, the cell types in hardwoods are illustrated. In the axial direction of the tree trunk, ≈1 mm long fibers provide mechanical support; wider vessels elements primarily provide water transport. In balsa, the vessels are ≈ 280 µm wide and homogenously distributed. Fibers are about 40 µm in diameter with 1.5 µm thick cell walls and empty lumen space in the central region (**Figure** **2**a). The cell wall of native wood is arranged as nanocomposite layers where cellulose fibrils are aligned and embedded in a mixed hemicellulose and lignin matrix (see inset illustration in Figure 1d). Lignin is a biopolymer containing light‐absorbing chromophores, contributing to brownish wood color. The wood compositions of balsa, alder, birch, and beech are detailed in Table S1, Supporting Information.

In order to prepare TW, a green and selective delignification method was used, based on peracetic acid (PAA) for lignin removal.^[^ ^45^, ^46^ ^]^ After PAA delignification, the resulting DW substrate appears white with retained cell structure, as shown in Figure 2b. The effects of delignification at micro‐scale showed as formation of pores in the lignin‐rich regions at cell corners and in the middle lamella between cells (see inset illustration in Figure 1d). The formation of nanoscale pores in the cell wall is also apparent in the high‐resolution SEM image in Figure 2b. The observation of increased porosity is supported by sharp increase in BET specific surface area (*S*_BET_) from 2 to 259 m^2^ g^−1^ for balsa substrate. The high surface area of this DW substrate is advantageous for chemical functionalization, since cell wall accessibility should be increased, facilitating reactant diffusion into the cell wall.

In a second stage, succinylation was performed on DW substrates by a recently developed green procedure,^[^ ^47^ ^]^ to reduce moisture sensitivity and facilitate monomer impregnation. Cell wall modification was successful when DW was acetylated and impregnated by methyl methacrylate (MMA) monomer.^[^ ^7^ ^]^ In the present method, the introduced SA carboxyl groups in DW‐SA also have the potential to form covalent ester linkages with the *β*‐hydroxyl groups of the PLIMA repeat unit. The DW substrates were esterified under solvent‐free conditions during short reaction times (30 min) using SA from renewable resources. The SA was derived from bio‐based succinic acid via ring‐closing dehydration (see ^1^H and C^13^ NMR spectra in Figure S4, Supporting Information). The succinylated wood substrate (DW‐SA) is colorless and structurally preserved (Figure 2c). During succinylation, the wood cell wall hydroxyls ring‐open SA to form both an ester and a free carboxylic acid. The FTIR spectra in Figure S5a, Supporting Information, confirm the successful attachment of SA to the DW substrate. After succinylation, the intensity of the peak at 1725 cm^−1^ associated with C=O stretching vibration, is considerably increased because of the carboxyl groups from a successful SA attachment. The covalent attachment of SA to the cell wall was confirmed by deprotonation of the terminal carboxylic acids to their carboxylate form. Deprotonation results in two distinct carbonyl peaks in the FTIR spectrum, associated with the ester carbonyl and sodium carboxylate. Moreover, the moisture sorption of the wood substrate is reduced after succinylation (Figure S5b, Supporting Information). This is attributed to the bulking effect from SA molecules in the cell wall (occupying space available for moisture).^[^ ^47^ ^]^

LIMA infiltration and subsequent polymerization into PLIMA in the wood substrate results in a biocomposite with preserved wood morphology. Observation of TW and TW‐SA SEM micrographs (Figure 2d and Figure S6, Supporting Information) demonstrates the successful impregnation of PLIMA into the vessels and lumen space at the center of the fiber cells. Possibly, the *β*‐hydroxyl groups in the repeat unit of PLIMA contribute to favorable secondary interactions with the wood substrate. The interactions should be further improved in TW‐SA. The residual moisture content is lowered,^[^ ^47^ ^]^ and in analogy with acetylation treatment, the tendency for cellulose fibril agglomeration should be decreased.^[^ ^8^ ^]^ In addition, the terminal carboxyls introduced in DW‐SA can covalently link with PLIMA. A related observation is the formation of covalent linkages between carboxyls on TEMPO‐CNF and hydroxyl groups.^[^ ^48^ ^]^ The covalent attachment between SA and LIMA was confirmed by ^13^C NMR after a model reaction using LIMA and succinic acid. The ^13^C NMR spectra reveal the formation of a new ester peak (see the detailed spectra in Figures S7 and S8, Supporting Information), which is direct evidence of covalent attachment via ester formation. The mechanism of ester formation between DW‐SA carboxyl groups and the LIMA *β*‐hydroxyl groups is detailed in Schemes S2 and S3, Supporting Information. Since these links are readily detected in the model reaction, it supports the formation of covalent linkages between DW‐SA and PLIMA in the wood cell wall.

In TW biocomposites, the PLIMA polymer matrix is located in the pore space of the DW and DW‐SA substrates. PLIMA has several functions in the biocomposite, including reduced hydrophilicity and moisture sorption, and improved thermal stability. Here, PLIMA and the bio‐based TW are water‐resistant and the photographs in Figure S9a,b, Supporting Information, illustrate water resistance of neat PLIMA polymer and the completely bio‐based TW composites. The static water contact angle of PLIMA (66°) is lower than for PMMA (72°), suggesting that LIMA monomer may show better interactions with the wood substrate. The composite morphology includes a thin surface layer (≈100 µm) of PLIMA which prevents water from direct contact with wood fibers; similar contact angles of 68° and 69° were measured for TW and TW‐SA biocomposites, respectively (Figure S9c, Supporting Information).

PLIMA and the bio‐based TW demonstrate suitable thermal stability for a wide range of applications. The thermal stability of PLIMA and biocomposites was evaluated using thermogravimetric analysis (TGA) in nitrogen. Note that succinylation of the DW substrate reduces thermal stability because of the carboxylic acid groups added (Figure S10a, Supporting Information). The TGA results show that thermal degradation of neat PLIMA is initiated at 207 °C (Figure S10b, Supporting Information). After LIMA impregnation of the delignified wood reinforcements and polymerization to PLIMA, the thermal stability of the TW and TW‐SA biocomposites is increased compared with neat PLIMA, and degradation is initiated at 230 °C.

### 2.3 Cell Wall Nanostructure

The nanostructure of the cell wall and wood‐polymer interphase region in TW are fundamental to biocomposite materials' properties. The question is if the LIMA monomer, and the present TW preparation protocol, can be used to have PLIMA matrix also inside the cell wall of the wood substrate. High‐resolution micrographs of delignified wood substrates in Figure 2b,c indicate pores at the 100 nm scale (compare with native wood, Figure 2a). Possibly, the TW‐SA biocomposite in Figure 2d indicates reduced porosity, and the presence of polymer inside the cell wall, surrounding microfibrils.

**Figure 3:**
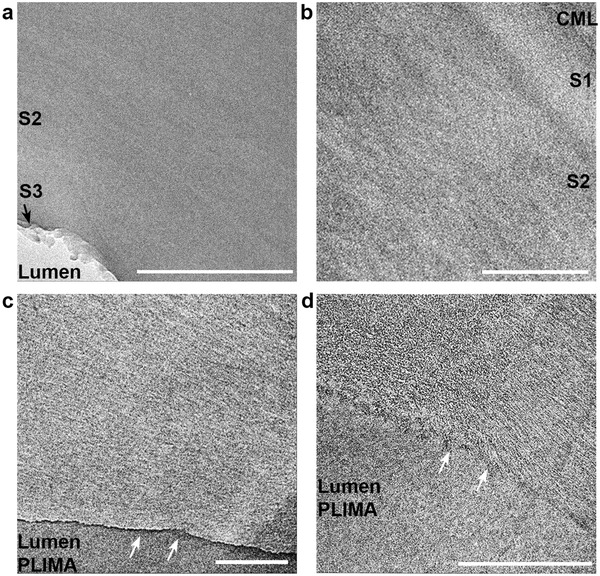
TEM images from a cross‐section of a) DW and b) DW‐SA cell wall. The top region corresponds to the compound middle lamella (CML), followed by the secondary cell wall layers S1 (outer), S2, and S3 (inner). Cross‐sections of the c) TW and d) TW‐SA biocomposites, where the arrows show the interface between the cell wall and the lumen filled by PLIMA. The scale bars correspond to 500 nm.

To assess the nature of the cell wall in the polymer matrix TW biocomposites, we prepared cryo‐ultrathin sections followed by TEM analysis. **Figure** **3**a–d shows the cell wall nanostructure of the porous DW and DW‐SA substrates, as well as the TW and TW‐SA biocomposites. The PLIMA‐impregnated TW and TW‐SA biocomposites in Figure 3c,d show apparent fibrillar structures at nano‐scale. The TEM micrograph of TW‐SA in Figure 3d clearly exhibits elongated fibrillar structures of a few nanometers in width, which are cellulosic fibrils. The PLIMA phase was subjected to osmium staining targeting the double bonds in PLIMA. Since the cellulose fibrils in the TW biocomposites show much higher contrast than cellulose fibrils in the DW and DW‐SA substrates (Figure 3a,b), this is direct evidence that the PLIMA polymer is located inside the cell wall. Micrographs are in support of truly nanostructured composites in the cell wall regions, with cellulose fibrils in a matrix of PLIMA and other components in the DW substrates (hemicelluloses, succinic anhydride).

No cellulose fibril aggregates are visible at this scale, suggesting that the polymer is well distributed (Figure 3c,d). Thus, the diffusion of LIMA monomer into the cell wall is facilitated by the nanoporosity after delignification, and succinylation. Impregnation of LIMA monomer was observed to be faster for the succinylated DW‐SA substrate, compared with the unmodified DW.

To further support the presence of PLIMA in the cell wall, a model experiment using energy dispersive X‐rays spectroscopy (EDS) analysis was performed. Fluorinated‐LIMA monomer was synthesized (Figure S11a, Supporting Information) to prepare fluorinated TW and TW‐SA biocomposites. Cross‐sectional EDS maps of the fluorinated TW biocomposite show that fluorine atoms are homogenously distributed inside the material (Figure S11b, Supporting Information). At the cell wall scale, EDS maps reveal the presence of fluorine atoms inside the cell wall of both TW and TW‐SA (Figure S11c,d, Supporting Information). The fluorine content was higher in fluorinated TW‐SA (7.6 wt%) than TW (5.9 wt%), due to faster diffusion of fluorinated LIMA in succinylated substrates. These results correlate with TEM observations and confirm the diffusion of LIMA into the cell walls of both TW and TW‐SA.

TEM micrographs show variations in the lumen‐cell wall interphase regions of TW (Figure 3c) and TW‐SA (Figure 3d), see also Figure S12, Supporting Information. Indeed, the interface between the wood cell wall and PLIMA in the unmodified TW cross‐section is clearly visible with a sharp boundary. In the case of TW‐SA, the interphase seems much more smooth and well‐integrated. Factors such as lower residual moisture content, better compatibility, and covalent bonds between PLIMA in lumen space and the SA‐modified wood cell wall may contribute. Variations in the lumen‐cell wall interphase region are likely to be critical for optical properties of the biocomposites.

### 2.4 Optical Properties

The optical properties (transmittance and haze) depend on scattering effects at the lumen‐cell wall interfaces between wood and PLIMA, but also on scattering inside the cell wall (Rayleigh scattering at the nanoscale) and the presence of optical defects such as voids. High optical transmittance of a two‐phase biocomposite certainly relies on good matching of refractive indices of the wood reinforcement and the PLIMA matrix, which reduces light scattering. Here, the newly synthesized PLIMA polymer showed a measured refractive index of 1.52 in the visible wavelength range, which is very close to that of the DW substrate (1.54)^[^ ^43^ ^]^ and this contributes to limited scattering effects.

**Figure 4:**
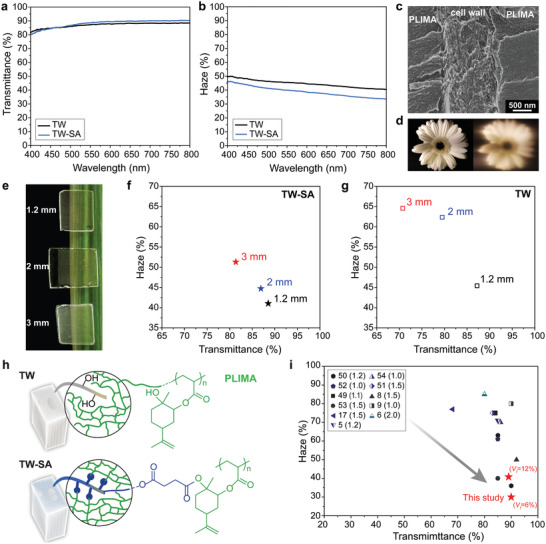
Optical properties of the sustainable transparent wood composites showing the a) transmittance and b) haze of 1.2 mm thick TW and TW‐SA prepared from balsa with a wood volume fraction (*V*_f_) of 12%. c) SEM micrograph of TW‐SA showing the interphase region between PLIMA and cell wall. d) Photographs taken at a distance of about 20 cm from the flower before (left) and after (right) placing a 1.2 mm thick TW‐SA sample in front of the camera lens. e) Photographs of TW‐SA composites prepared from balsa of various thicknesses. Optical haze versus transmittance of 1.2, 2, and 3 mm thick f) TW‐SA and g) TW biocomposites prepared from balsa (*V*_f_ = 12%) at a wavelength of 550 nm. h) Illustration showing the interactions between wood substrate and PLIMA in TW and TW‐SA. i) Haze versus transmittance for documented TW composites of similar thickness, compared to this study (1.2 mm thick TW‐SA based on balsa, *V*_f_ = 6% and 12%).^[^ ^5^, ^6^, ^8^, ^9^, ^17^, ^49^, ^50^, ^51^, ^52^, ^53^, ^54^ ^]^ Numbers in parentheses represent the thickness of the sample.

Succinylation results in a TW‐SA biocomposite combining high optical transmittance with exceptionally low haze (forward scattered light), see **Figure** **4**. The optical transmittance of TW‐SA at 550 nm is 89%, which is higher than for TW (87%) as shown in Figure 4a. Haze data (transmitted but scattered light) for the present TW materials are remarkably low (Figure 4b). Succinylation reduces light scattering and haze in TW‐SA so that haze (41%) becomes lower compared with TW (46%). The decrease in haze is attributed to a lower amount of nanostructural defects in the cell wall, thereby reducing Rayleigh scattering at the nanoscale, although one cannot exclude contributions from lumen‐cell wall interface defects. TW‐SA shows lower porosity (≈ 0% versus ≈ 1% for TW) and the molecular scale interaction between DW‐SA and PLIMA is likely to be improved, compared with unmodified TW. Thus, the remarkable optical properties of TW‐SA are the result of the combination of enhanced compatibility between PLIMA and wood cell wall (Figure 4c) with well integrated lumen‐cell wall interface, low extent of cellulose fibril aggregation, and favored diffusion of LIMA monomer inside the cell wall prior to polymerization (Figure 3d). The TW‐SA biocomposite shows an optically clear appearance. This is demonstrated in Figure 4d, where the features of the underlying image are visible through the TW‐SA material.

The difference in optical transmittance between TW and TW‐SA increases with thickness. The improvement for TW‐SA at thicknesses of 1.2, 2, and 3 mm are visible in the photographs in Figure 4e and Figure S13, Supporting Information, where the background image and letters are apparent. In general, increase in wood substrate thickness results in greater light attenuation, and thereby lower transmittance, because of larger absorption as well as a larger number of defects acting as scattering sites.^[^ ^49^ ^]^ Here, succinylation results in maintained high transmittance and limited decrease in haze (Figure 4f). At a thickness of 3 mm, TW‐SA shows high transmittance (81%) and a low haze of 51%. In contrast, TW demonstrates much lower optical transmittance (71%) and higher haze (65%) at similar thickness (Figure 4g). The reduced light scattering effects in thick TW‐SA compared with the TW reference are ascribed to a favorable distribution of the refractive index‐matched PLIMA in the cell wall and well‐integrated interface between wood components and PLIMA (Figure 4h).

Compared with data for other reported TWs prepared from petroleum‐based polymer matrices, the present TW‐SA is a sustainable and bio‐based alternative with a remarkably low haze and high transmittance (Figure 4i).

### 2.5 Mechanical Performance and Materials Design

**Figure 5:**
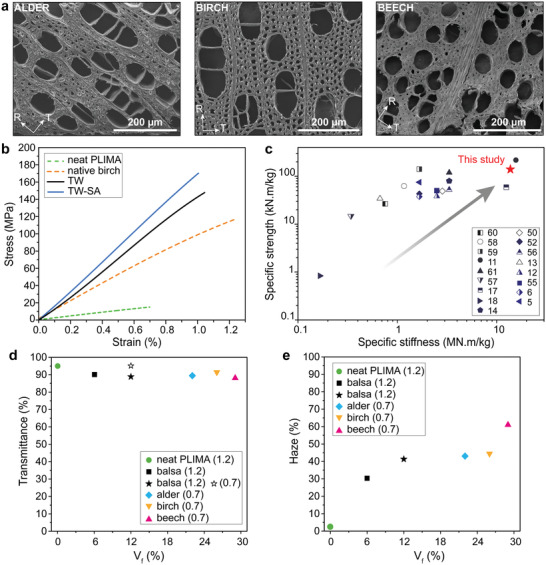
a) SEM micrographs of alder, birch, and beech wood species. b) Mechanical properties of neat PLIMA, native birch, and TW and TW‐SA biocomposites prepared from birch. c) Specific strength versus specific stiffness for documented TW composites, compared to this study (TW‐SA prepared from birch).^[^ ^5^, ^6^, ^11^, ^12^, ^13^, ^14^, ^17^, ^18^, ^50^, ^52^, ^55^, ^56^, ^57^, ^58^, ^59^, ^60^, ^61^ ^]^ d) Transmittance of TW‐SA biocomposites prepared from various wood species as a function of wood volume fraction (*V*_f_). The transmittance of 0.7 mm thick balsa (*V*_f_ = 12%) corresponds to the predicted value. e) Haze of TW‐SA biocomposites prepared from various wood species as a function of *V*_f_.

The optical and mechanical properties of TW biocomposites depend on wood volume fraction and characteristics of the wood‐polymer interface. A higher wood content improves mechanical properties, but reduces optical transmittance. In order to assess the potential of fully bio‐based TW for high performance applications, biocomposites were prepared from higher density wood species (Table 1). We successfully performed succinylation on alder, birch, and beech wood species of different chemical compositions (Table S1, Supporting Information), densities (Table 1), and morphologies (**Figure** **5**a). A high degree of esterification is obtained even for high‐density beech wood, as indicated by the large increase in carboxyl intensity in the FTIR spectra after succinylation (Figure S14, Supporting Information).

In order to reach high axial biocomposite strength and Young's modulus, high wood volume fraction, low porosity, and strong interfacial adhesion between the matrix and the reinforcement phase are essential. Birch with a wood volume fraction of 26% was therefore used to evaluate longitudinal tensile properties. Figure 5b shows the stress–strain curves of neat PLIMA, TW, and TW‐SA, and the mechanical properties are summarized in **Table** **2**. Even without the SA‐treatment, the PLIMA matrix shows good mechanical function and TW reaches an ultimate longitudinal strength *σ*_c_ of ≈147 MPa and a Young's modulus *E*_c_ of ≈13 GPa, which is higher than for native birch. The succinylation treatment (SA) results in even better mechanical properties, see Figure 5b. The properties of TW‐SA surpass those of TW with ultimate strength *σ*_c_ of TW‐SA ≈174 MPa and Young's modulus *E*_c_ ≈17 GPa. These data are better than for most reported TW biocomposites from petroleum‐based polymer matrices (Figure 5c).

**Table 2:** Density, wood volume fraction (*V*_f_), and mechanical properties in tension of neat PLIMA, native birch, TW, and TW‐SA prepared from birch

	Density [kg m^−3^]	*V*_f_ [%]	Ultimate strength [MPa]	Young's modulus [GPa]	Strain to failure [%]	Effective strength *σ*_f_ [MPa]	Effective modulus *E*_f_ [GPa]
PLIMA	1134	0	14.9 ± 6.6	2.2 ± 0.4	0.7 ± 0.3		
Native birch	593	40	116.4 ± 16.4	10.6 ± 2.4	1.2 ± 0.3	293	27
TW	1229	26	146.6 ± 17.9	12.6 ± 1.7	1.2 ± 0.4	523	42
TW‐SA	1240	26	173.6 ± 14.5	17.3 ± 4.5	1.0 ± 0.2	623	60

An excellent way of comparing cell wall properties in wood with those in the two composites is to estimate effective reinforcement modulus *E*_f_ and strength *σ*_f_ for all materials. The procedure is explained in the Supporting Information. The starting point is the effective birch cell wall modulus (*E*_f_ = 27 GPa) and tensile strength (*σ*_f_ = 293 MPa), see Table 2. It is quite interesting that effective cell wall properties are strongly improved for the TW biocomposite based on unmodified wood substrate. The modulus *E*_f_ goes from 27 to 42 GPa and the strength *σ*_f_ from 293 to 523 MPa. The main reason for this is probably local inhomogeneities such as the uneven cell wall thickness of wood fibers, combined with the marginal contribution of lignin to tensile stiffness and strength at the cell wall level. It means that the cell wall in native wood has an uneven stress distribution at the microscale, with regions of low stress, and regions of much higher stress. The PLIMA polymer matrix results in a more homogeneous stress distribution in the cell wall, and the net result is an improved stiffness contribution from the wood cell wall. Apparently, the effect is even stronger for strength.

For the TW‐SA biocomposite, with modified wood cell wall, the effective cell wall properties are even better. Compared with TW, the effective modulus *E*_f_ goes from 42 to 60 GPa, an improvement of almost 50%. Because of this strong improvement, the succinylation somehow contributes to cell wall modulus and improves stress transfer between cellulose fibrils. The effective strength *σ*_f_ also improves, from 523 to 623 MPa with succinylation. One may note that fracture surfaces indicate shorter wood fiber pull‐out lengths in TW‐SA, which should be related to stronger interfacial adhesion between PLIMA and the wood cell wall (Figure S15, Supporting Information).

For materials design purposes, we compared the optical properties of biocomposites from balsa, alder, birch, and beech, with different wood contents. Interestingly, succinylation results in biocomposites with higher optical transmittance for all wood species (Figure 5d and Table S2, Supporting Information). The present succinylation method is thus applicable to a wide range of wood species for improved nanostructural control. The optical haze data of these biocomposites are presented in Figure 5e. If the balsa wood volume fraction is reduced to 6% in TW‐SA, the haze is lowered to 30%. The decrease is attributed to fewer scattering sites associated with reduced wood volume fraction. For comparative purposes, we predicted the transmittance values of balsa‐based biocomposites at 0.7 mm thickness based on the model by Chen et al.^[^ ^49^ ^]^ The values are 92.3% for TW and 95.1% for TW‐SA (see details in Figure S16, Supporting Information). Note that the predicted transmittance for 0.7 mm balsa‐based TW‐SA is very high and similar to neat PLIMA. The optical transmittance of TW‐SA was reduced from 95% to 88% with increased wood volume fraction from 12% (balsa) to 29% (beech) because of increased scattering effects. The generally high transmittance and low haze of TW‐SA from alder, birch, and beech are demonstrated in the photographs in Figure S17, Supporting Information.

Therefore, the present method for fully bio‐based and transparent biocomposites with tailored wood‐polymer interface is applicable to a wide range of wood species, enabling tunable optical and mechanical performance depending on the application. For higher mechanical performance, wood species of higher density are desirable, such as birch or beech. Lower density wood species, such as balsa, are more favorable for high optical transmittance and low haze.

## 3 Conclusion

A novel, bio‐based LIMA monomer based on a food industry waste product, was designed, and prepared by green synthesis. It was impregnated in delignified wood substrates, without solvent, and polymerized to form fully bio‐based transparent wood biocomposites. In order to remove any moisture sorption problems and facilitate nanoscale monomer cell wall impregnation, the wood substrate was successfully modified by bio‐based succinic anhydride (SA) using a green process. The fully bio‐based transparent wood showed excellent mechanical properties for load‐bearing functions, combined with high optical transmittance and low haze. The reason is wood‐PLIMA matching of refractive indices, a very low content of optical defects and a well‐integrated interface between PLIMA polymer and cellulosic wood. SA succinylation of wood improves interface interactions and facilitates monomer impregnation significantly. After succinylation of the wood substrate, the free carboxyl binds covalently to the *β*‐hydroxyl designed into the LIMA monomer. The effective reinforcement properties are strongly improved in succinylated TW‐SA, where strong interfacial adhesion between PLIMA and the wood cell wall results in biocomposites of high mechanical performance.

This fully bio‐based composite with optical transmittance function is a sustainable alternative for engineering applications. Sustainable material components, processes, and protocols for molecular and nanostructural control have been developed. Further functionalization by nanoparticles, dyes, or additives for advanced photonics applications can be readily performed. A remaining challenge is to find processing concepts suitable for larger structures with controlled micro‐ and nanostructure, so that sustainable wood nanotechnologies can compete with translucent plastics and glass in applications exemplified by load‐bearing panels, in interior design, lighting, and various energy‐saving purposes (heat‐storage, thermal insulation, controlled use of natural light, etc.).

## 4 Experimental Section

##### Materials

Balsa (*Ochroma pyramidale*) veneers with oven‐dried densities of 103 and 304 kg m^−3^ were purchased from Material AB (Sweden). Silver birch (*Betula pendula*) with an oven‐dried density of 593 kg m^−3^ was purchased from Glimakra AB (Sweden). Alder (*Alnus glutinosa*) with an oven‐dried density of 510 kg m^−3^ and beech (*Fagus sylvestris*) with an oven‐dried density of 631 kg m^−3^ were purchased from Calexico Wood AB (Sweden). The bio‐based succinic acid was donated by Matsen Chemie AG (Germany). Acetone (≥99.5%) was purchased from VWR. The peracetic acid (PAA, 40%), ethyl acetate (EtOAc, ≥99.5%), succinic anhydride (SA, ≥99%), acrylic acid (99%), 4‐methoxyphenol (≥98.0%), limonene oxide (97%), sodium carbonate (Na_2_CO_3_, ≥99.5%), and 2,2′‐azobis(2‐methylpropionitrile) (AIBN, 98%) were purchased from Sigma Aldrich and used as received.

##### Delignification and Succinylation of the Wood Substrate

The thickness of the wood veneers was of 0.7 mm for alder, birch, and beech wood species. The balsa wood veneers were 1.2, 2, and 3 mm thick. Delignification was performed using an aqueous PAA solution (4 wt%) at a pH of 4. 8 (adjusted with sodium hydroxide). The delignification was left to proceed at 80 °C under stirring until the samples became white. After the delignification, the samples were washed several times with deionized water and solvent exchanged to acetone. The delignified wood (DW) samples were kept in acetone until further use. Succinylation was performed via ring‐opening esterification reaction of SA on the DW substrate in accordance to our previous protocol.^[^ ^47^ ^]^ The ring‐opening esterification reaction was performed on DW under neat conditions for 0.5 h at 130 °C without stirring. After the reaction, the DW samples were thoroughly washed with acetone five times to remove any non‐bonded reagent. The DW substrate functionalized with SA is abbreviated as DW‐SA. The renewable succinic acid was converted to the anhydride form using distillation equipment, and by heating at 200 °C under stirring.

##### Ring‐Opening Acrylation of Limonene Oxide with Acrylic Acid

The desired amount of limonene oxide (21.4 g, 0.14 mol, 1 equiv.) and acrylic acid (40 g, 0.55 mol, 4 equiv.) and 4‐methoxyphenol (0.69 g, 0.0055 mol, 0.04 equiv.) was added to a 100 ml round bottom flask equipped with a magnetic stirrer. The reaction mixture was bubbled with Ar for 5 min at ambient temperature to remove any residual oxygen. The round bottom flask was capped and immersed in an oil bath set at 75 °C for 3 h. After the reaction the mixture was cooled to ambient temperature, decanted into 500 ml beaker with 200 ml deionized water and 200 ml of EtOAc. Under stirring Na_2_CO_3_ was added portion wise to the mixture until no more CO_2_(g) was formed. After deprotonation of acrylic acid, the mixture was poured in a separation funnel and the EtOAc phase was isolated and washed three times with NaHCO_3_ (sat.), and finally two times with 10 wt% NaOH. The EtOAc phase was dried with MgSO_4_(s) and concentrated to yield the desired product limonene acrylate (LIMA) as a transparent oil (27.3 g, 0.12 mol, 87% yield).

##### Preparation of the Sustainable Transparent Wood Biocomposites

DW substrates were infiltrated under vacuum for 2 h (6 h for 3 mm thick samples) with a mixture of LIMA monomer and 0.5 wt% of AIBN as initiator. The infiltrated samples were then placed between two glass slides, packaged in aluminum foil, and polymerized at 75 °C for 24 h. The resulting transparent wood biocomposites are termed as TW, and the transparent wood functionalized with SA as TW‐SA.

##### Characterization

A Spectrum 100 Fourier transform infrared (FT‐IR) spectrometer (PerkinElmer, USA) equipped with a Golden Gate diamond ATR (Gaseby Specac Ltd, UK) was used to assess the effects of the chemical modifications on the wood substrate. The spectra were recorded at room temperature with a resolution of 4 cm^−1^. The lignin content of the native and delignified wood substrates was determined by the Klason lignin method, and carbohydrate analysis was performed using a Dionex ICS‐3000 ion chromatography system (Thermo Fisher Scientific, USA). The monosaccharides determined are arabinose, galactose, xylose, mannose, and glucose, where the cellulose content was assumed equal to the glucose content. Nuclear magnetic resonance (NMR), ^1^H, and ^13^C NMR, results were recorded at room temperature on a Bruker Avance III HD 400 MHz instrument with a BBFO probe equipped with a *Z*‐gradient coil for structural analysis. Data were processed with MestreNova (Mestrelab Research) software using a 90° shifted square sine‐bell apodization window, baseline and phase correction were applied in both directions. The samples morphologies were acquired with a field‐emission scanning electron microscope (FE‐SEM, Hitachi S‐4800, Japan). All samples were Pt/Pd sputtered under vacuum (Cressington R208, UK). The delignified wood cross‐sections were obtained by sectioning the wood samples with a microtome (Leica SM2010 R Sliding Microtome, Germany) in the wet state, followed by drying with supercritical CO_2_ (Autosamdri‐815, Tousimis, USA). The transparent wood composites cross‐sections were prepared using an ultramicrotome (MT‐XL, RMC, UK) at room temperature equipped with a glass knife. EDS mapping and point measurements were carried out using SEM equipped with an X‐Max 80mm^2^ EDS detector (Oxford Instruments, UK) at an accelerating voltage of 5 kV. Cryo‐ultramicrotomy and transmission electron microscopy were performed for cell wall analysis. Ultrathin sections of transparent and delignified wood were prepared at low temperature (−20 to −90 °C) using an Ultracut UC6 microtome and an FC7 cryo‐chamber (Leica Microsystems GmbH, Austria) with a 35° dry cryo diamond knife (DiATOME, Switzerland). The sections were collected on glow‐discharged carbon‐coated copper grids. They were subsequently freeze‐dried in a precooled vacuum drier to avoid ultrastructural alteration due to ice crystal growth.^[^ ^62^ ^]^ The sections were then exposed to osmium tetroxide vapor for 30 min. Transmission electron microscopy was performed using a JEM‐2100Plus (Jeol Ltd, Japan). All the electron micrographs were recorded using a Gatan Rio 16 camera (Gatan Inc., USA) using the SerialEM program.^[^ ^63^ ^]^ Water sorption experiments were carried out in a controlled‐humidity chamber at 22 °C. The relative humidity (RH) was sequentially set to 0%, 12%, 50%, 75%, and 94%, where 0% RH corresponds to the oven‐dry weight. Five samples were left at each RH for one week before weighing. Static contact angle measurements were performed in triplicate using a PGX+ contact angle goniometer (Pocket goniometer, Sweden) with a MilliQ water automatic dispenser. Nitrogen physisorption was carried out at 77 K using a MicroActive 3Flex 3500 surface area and pore size analyzer (Micromeritics, USA). Before measurements, the samples were dried using supercritical CO_2_ and degassed at 70 °C for 48 h on a Smart VacPrep degassing system (Micromeritics, USA). Specific surface area was calculated according to a multipoint Brunauer–Emmett–Teller (BET) method. The refractive index of the poly(limonene acrylate) (PLIMA) polymer was measured on a J457 Refractometer (Rudolph Research Analytical, USA). The TW were prepared with dimensions of 1.5 cm × 1.5 cm for optical measurement. Optical transmittance and haze of the samples were acquired with an integrating sphere in accordance to a previous method, measurements were performed in triplicate.^[^ ^5^ ^]^ The sample was set in front of an input port of the integrating sphere, a quartz tungsten halogen light source (model 66181, Oriel Instruments) with a strong, stable output mainly in the visible and NIR region was applied as the incident beam. The output light was collected through an optical fiber connected to the output port of the integrating sphere. The thermal stability of the samples was studied by thermogravimetric analysis (TGA, Mettler Toledo TGA/DSC1, Switzerland) with a heating rate of 10 °C min^−1^ under nitrogen atmosphere. The temperature at a weight loss of 5% was taken as the onset degradation temperature. Dynamic mechanical thermal analysis (DMTA) was carried out using a DMA Q800 (TA Instruments, USA). Specimens of 20 × 5.0 × 0.5 mm^3^ were subjected to a strain of 0.05% of the sample length with a frequency of 1 Hz in tensile mode with a heating rate of 3.0 °C min^−1^. The *T*_g_ was taken at the maximum peak of the tan *δ* curve. The tensile properties of the samples were determined using a universal testing machine (Instron 5944, USA) equipped with a 2 kN load cell and a video extensometer. Five samples (5 mm × 5 cm) were preconditioned for 48 h at 50% RH before measurement. All samples were loaded in the longitudinal axis along the fiber direction. The tests were carried out at a temperature of 22 °C and 50% RH, with a 10% min^−1^ strain rate and span of 20 mm. The wood volume fraction (*V*_f_) was estimated from the DW substrate weight fraction (*W*_f_) following the equation: *V*_f_ *= (W*_f_ *× ρ*_c_ *)/ρ*_f_, where *ρ*_f_ is the cell wall density (1500 kg m^−3^) and the theoretical density of the biocomposite, *ρ*_c_, is equal to *ρ*_c_ = 1/(*W*_f_/*ρ*_f_ + *W*_m_ */ρ*_m_), with *W*_m_ and *ρ*_m_ the weight fraction and density of PLIMA, respectively. The porosity was calculated using the following equation: *ϕ* = (*ρ*_c_ − *ρ*_exp_)⁄*ρ*_c_, where *ρ*_exp_ is the experimental density of the biocomposite obtained with a pycnometer (Micromeritics AccuPyc 1330, USA).

##### Statistical Analysis

Quantitative data are expressed as mean and the sample size is given in the respective method description. The error was estimated through the calculation of the standard deviation (SD) of the mean and is given as mean ± SD.

## Conflict of Interest

The authors declare no conflict of interest.

## Supporting information

Supporting Information

## Data Availability

Research data are not shared.
