# Supplementary material for: High Performance, Fully Bio‐Based, and Optically Transparent Wood Biocomposites
Source: Adv Sci (Weinh). 2021 May 2;8(12):2100559. doi: 10.1002/advs.202100559 (PMC8224414; doi:10.1002/advs.202100559)
Supplement: Supplementary file 1 — Supporting Information [file ADVS-8-2100559-s001.pdf]

© 2021 Wiley-VCH GmbH

ADVANCED SCIENCE
Open Access

Supporting Information

for *Adv. Sci.*, DOI: 10.1002/advs.202100559

High Performance, Fully Bio-based, and Optically Transparent Wood Biocomposites

Céline Montanari, Yu Ogawa, Peter Olsén, and Lars A. Berglund**

Supporting Information

High performance, fully bio-based, and optically transparent wood biocomposites

Céline Montanari, Yu Ogawa, Peter Olsén, and Lars A. Berglund**

C. Montanari, Dr. P. Olsén, Prof. L. A. Berglund
Department of Fibre and Polymer Technology, Wallenberg Wood Science Center
KTH Royal Institute of Technology, Teknikringen 56, 100 44 Stockholm, Sweden
*E-mail: polsen@kth.se, blund@kth.se

Dr. Y. Ogawa
Univ. Grenoble Alpes, CNRS, CERMAV, 38000 Grenoble, France

Table of contents

Figures
Figure S1 2
Figure S2 3
Figure S3 4
Figure S4–5 5
Figure S6–7 6
Figure S8 7
Figure S9–10 9
Figure S11 11
Figure S12–14 12
Figure S15 14
Figure S16 16
Figure S17 17

Schemes
Scheme S1 3
Scheme S2–3 8

Tables
Table S1 4
Table S2 15

Methods
Fluorinated limonene acrylate synthesis and transparent wood preparation 10
Effective axial wood cell wall modulus E_f and tensile strength σ_f 13
Prediction of transmittance for balsa-based biocomposites 16

References 17

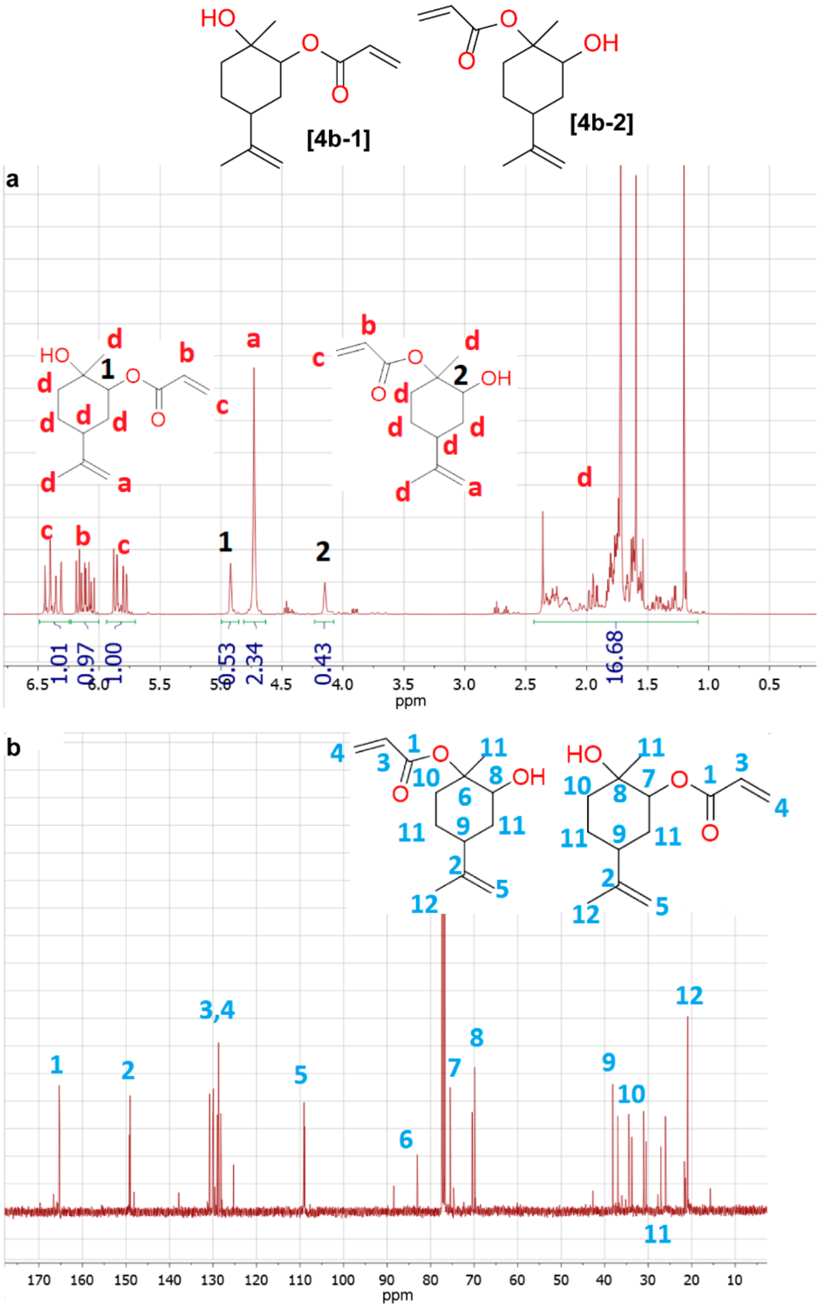

Figure S1. 2-hydroxy-2-methyl-5-(prop-1-en-2-yl)cyclohexyl acrylate (**[4b-1]**) and 2-hydroxy-1-methyl-4-(prop-1-en-2-yl)cyclohexyl acrylate (**[4b-2]**) in accordance to the described protocol, 33 g (yield 87 % as a transparent oil). a) ^{1}H NMR (400 MHz, Chloroform-d) δ 6.36 (ddd, J = 35.8, 17.3, 1.5 Hz, 1H), 6.10 (ddd, J = 31.3, 17.4, 10.4 Hz, 1H), 5.81 (ddd, J = 30.7, 10.3, 1.5 Hz, 1H), 4.91 (q, J = 3.2, 2.3 Hz, 0.55H), 4.73 – 4.70 (m, 2H), 4.14 (q, J = 3.5 Hz, 0.45H), 2.39 – 1.10 (m, 13H). b) ^{13}C NMR (101 MHz, Chloroform-d) δ 165.34, 165.20, 149.22, 149.03, 130.79, 130.03, 129.88, 129.02, 128.72, 128.21, 109.08, 108.94, 83.07, 75.49, 70.45, 69.87, 38.16, 36.99, 34.50, 33.77, 31.05, 30.51, 27.06, 26.08, 25.99, 21.68, 20.99, 20.92.

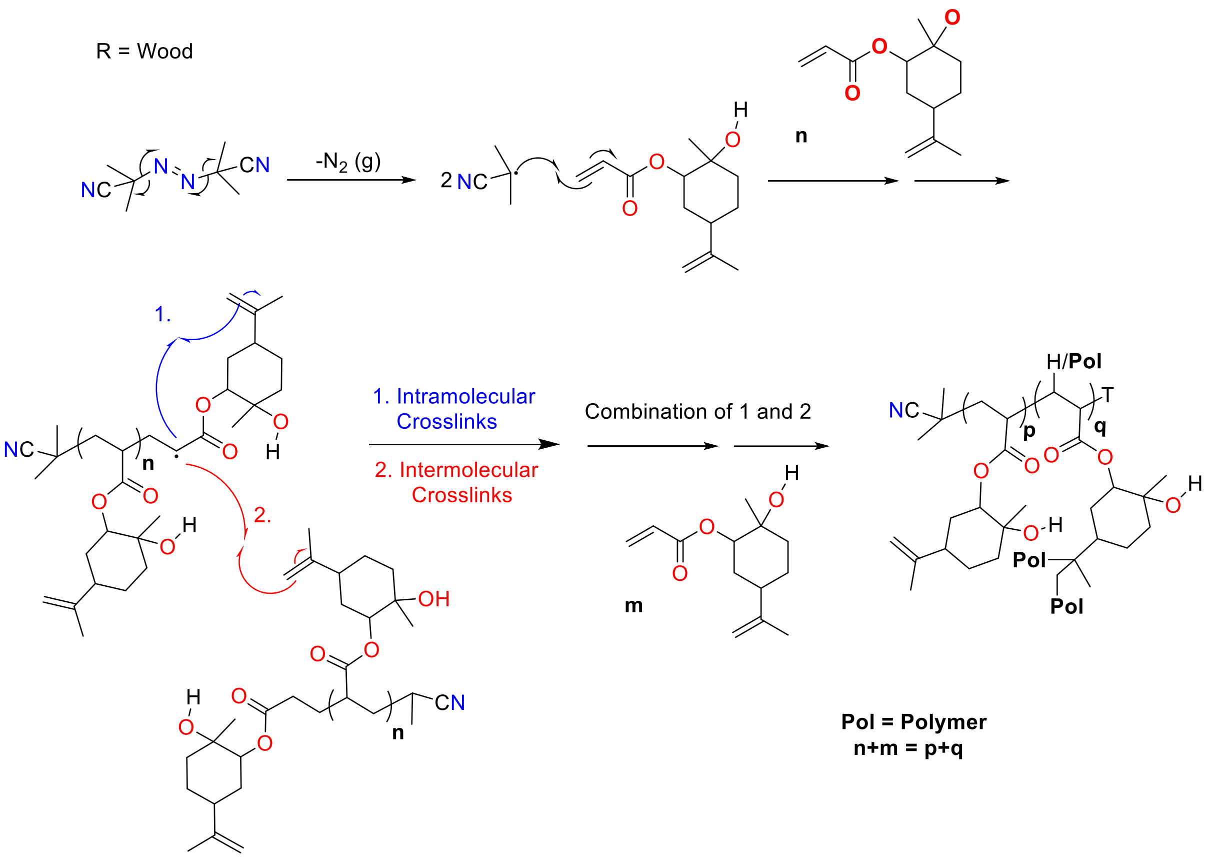

Scheme S1. Polymerization of LIMA and crosslinking mechanisms.

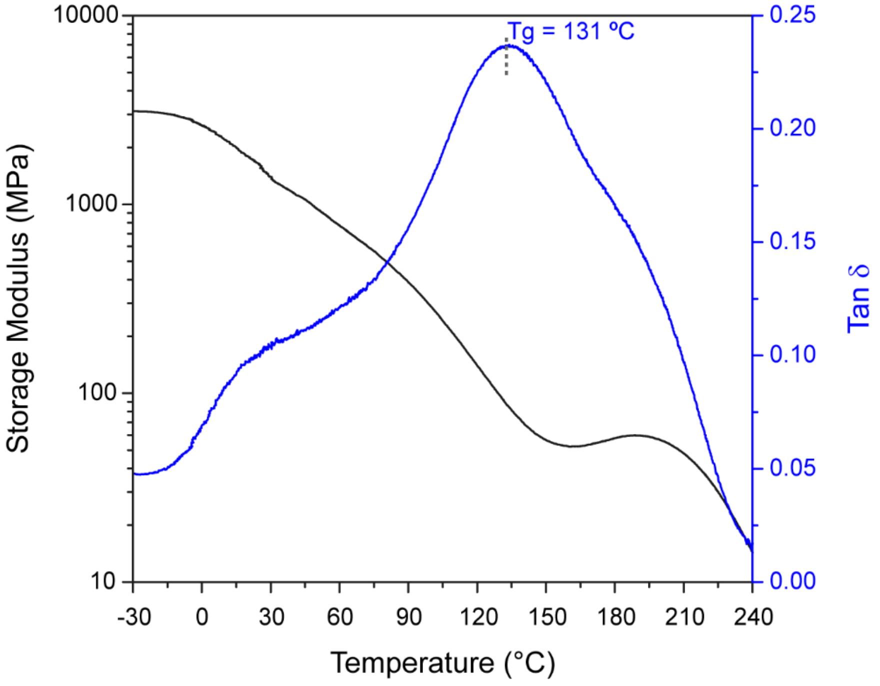

Figure S2. Storage modulus and mechanical damping factor tan δ of neat PLIMA as a function of temperature.

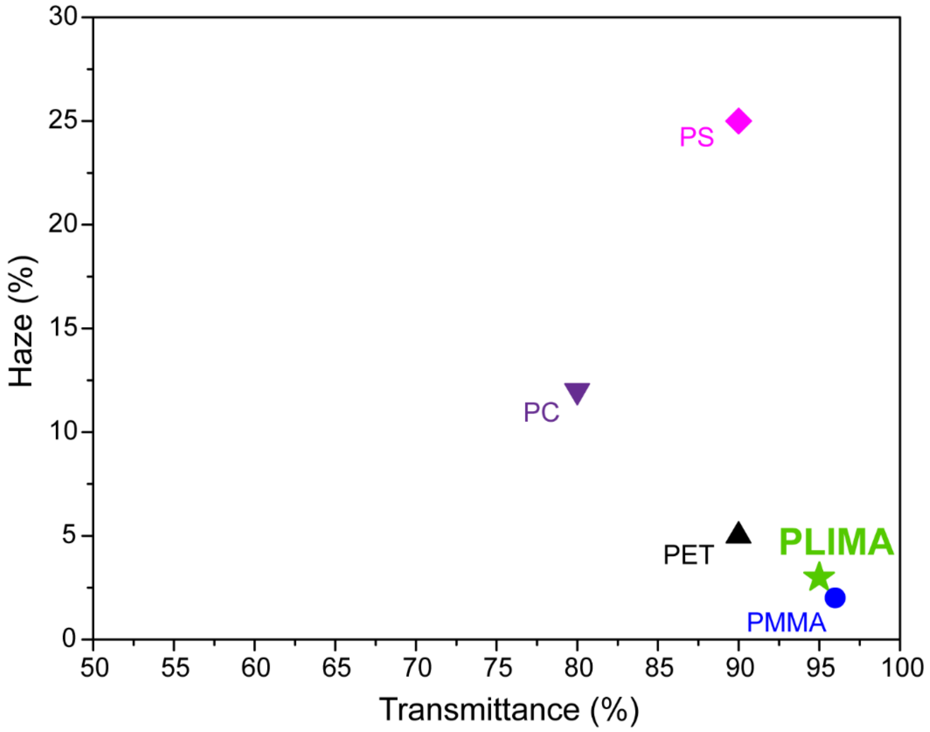

Figure S3. Optical properties of polystyrene (PS),[1] polycarbonate (PC),[2] polyethylene terephthalate (PET),[3] and polymethyl methacrylate (PMMA)[4] compared with bio-based PLIMA.

Table S1. Wood composition of various wood species used as reinforcement in TW and TW-SA composites.

		Lignin [%]	Cellulose [%]	Hemicellulose [%]
Balsa	NW	23.9	53.7	22.4
	DW	2.0	72.5	25.5
Alder	NW	27.9	47.0	25.1
	DW	1.2	72.7	26.1
Birch	NW	18.2	53.5	28.3
	DW	1.2	64.3	34.5
Beech	NW	21.7	50.3	28.0
	DW	0.9	66.6	32.5

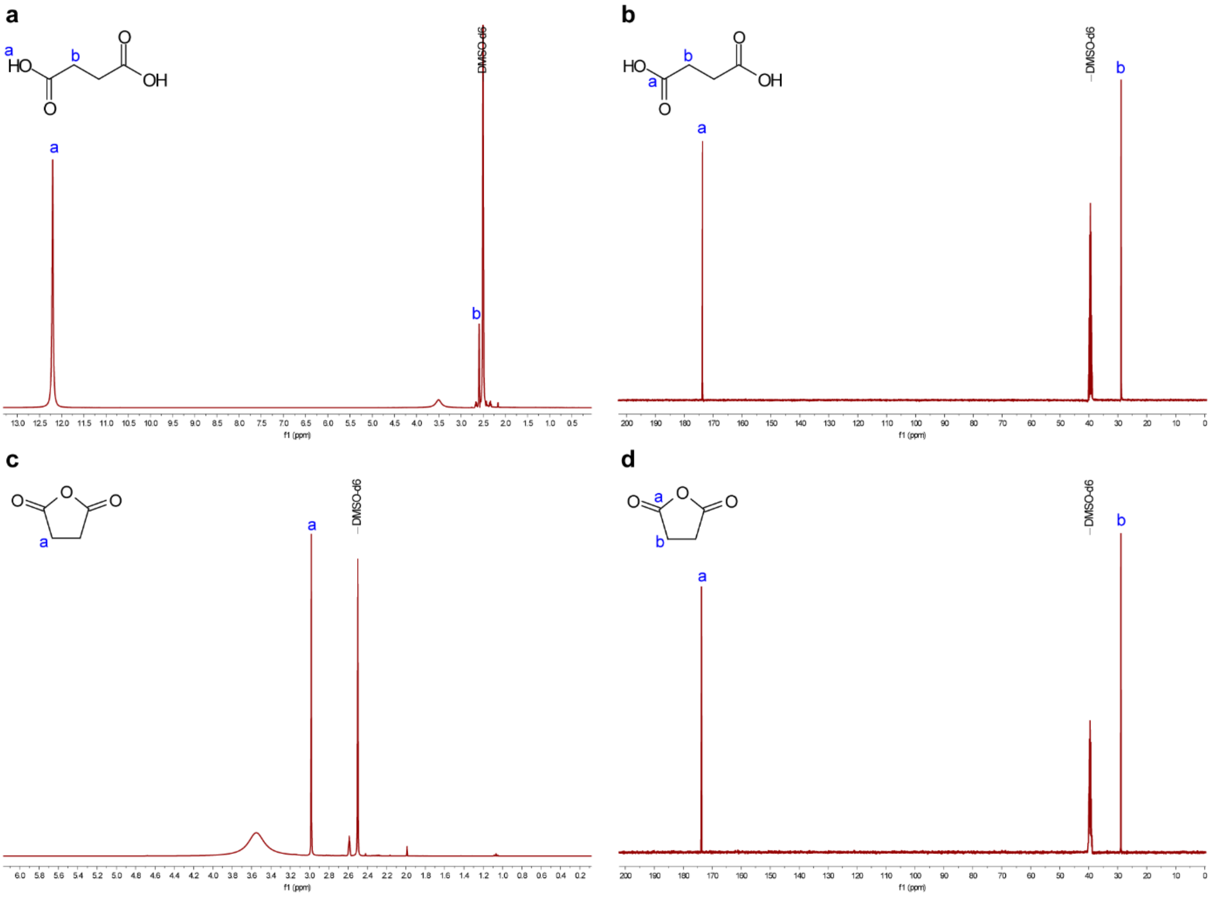

Figure S4. a) Succinic acid ^{1}H NMR (400 MHz, DMSO) δ 2.50 and (b) succinic acid ^{13}C NMR (101 MHz, DMSO) δ 39.18. c) Succinic anhydride ^{1}H NMR (400 MHz, DMSO) δ 2.50 and (d) succinic anhydride ^{13}C NMR (101 MHz, DMSO) δ 39.52.

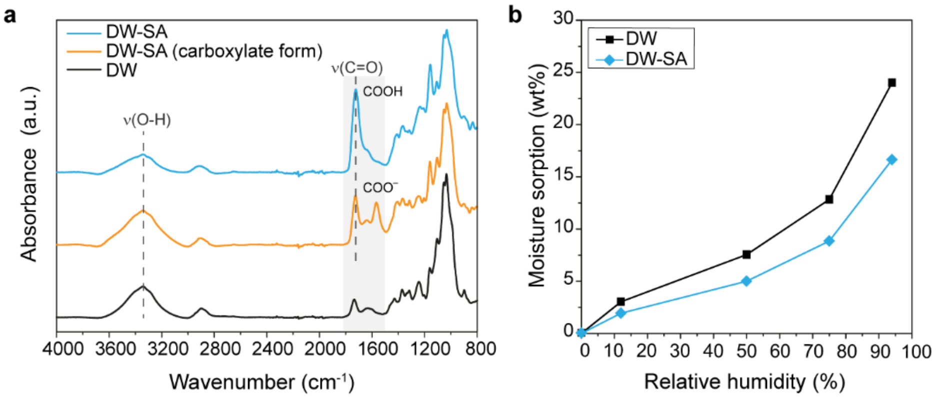

Figure S5. a) FTIR spectra of delignified wood (DW) and succinylated (DW-SA) substrates showing the carboxylate peak shift after deprotonation of the carboxyl group. b) Moisture sorption of DW and DW-SA measured at various relative humidities.

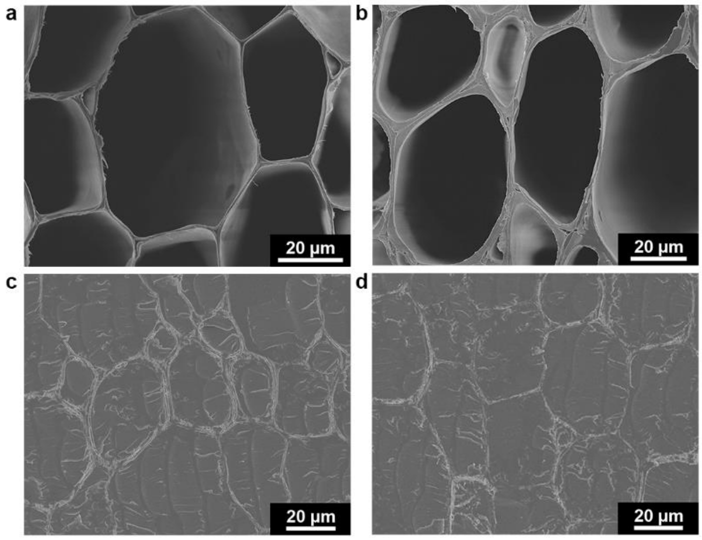

Figure S6. SEM images showing the wood cells of DW (a), DW-SA (b), TW (c), and TW-SA (d) samples.

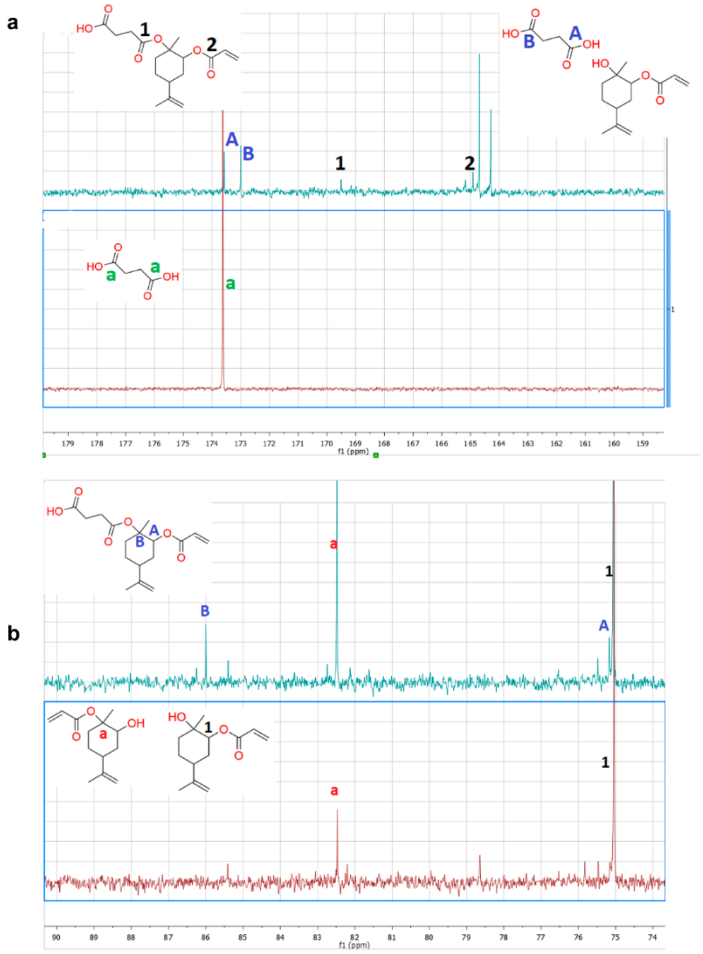

Figure S7. ^{13}C NMR (101 MHz, DMSO) spectra after reacting LIMA with succinic acid at 75 °C for 24 h under neat conditions. a) ^{13}C NMR at high shift carbonyl region, where the top-spectrum shows the product after reaction and the bottom spectrum is pure succinic acid.

b) ^{13}C NMR at alpha ester shift region, where the top-spectrum shows the product after reaction and the bottom spectrum is pure succinic acid.

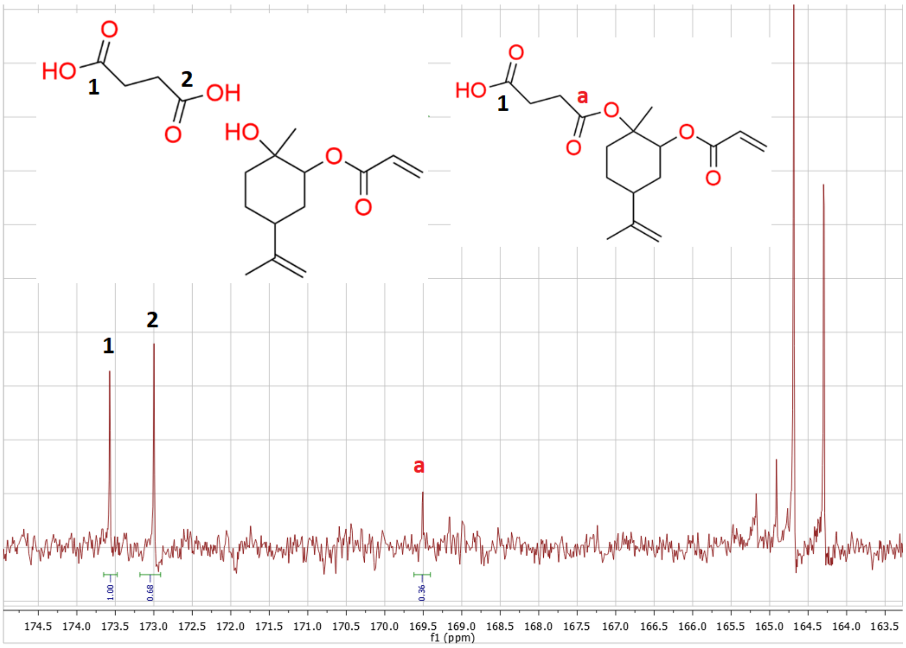

Figure S8. ^{13}C NMR (101 MHz, DMSO) at high shift carbonyl region after reacting LIMA with succinic acid at 75 °C for 24 h under neat conditions. The spectra was used to calculate the ester formation conversion, which was found to be 35 %.

To explore if covalent attachment occurs via esters formation between the succinylated DW-SA substrate and LIMA, we performed a model reaction between LIMA and succinic acid under neat conditions at 75 °C for 24 h. The molar ratio of succinic acid and LIMA was 1:1. The molecular changes that occurred was visualized by ^{13}C NMR. ^{13}C NMR is a powerful methodology to observe changes at a molecular level. After the reaction two significant molecular changes appear, a new carbonyl peak at high shift associated with the ester formation (Figure S7a), and the formation of a new peak alpha to ester (Figure S7b). After the reaction, it was found that the carbonyl peak associated with the free succinic acid splits into two different peaks, however, the shift was too high to indicate the formation of an ester. Instead, this indicates that the hydrogen-bonding environment of the carbonyl has changed. This was further verified by addition of triethylamine into the NMR sample, where only one peak at 175 ppm was shown. A new peak at 169.5 ppm and a peak at 86 ppm was observed after the reaction. These shifts correspond well to both the ester carbonyl and alpha proton next to the ester, see Figure S7. The calculated conversion towards ester after the reaction was found to be 35 % (Figure S8). The solubility of succinic acid in LIMA was poor under neat conditions; therefore, the actual conversion towards ester formation is believed to be even higher in TW-SA biocomposites.

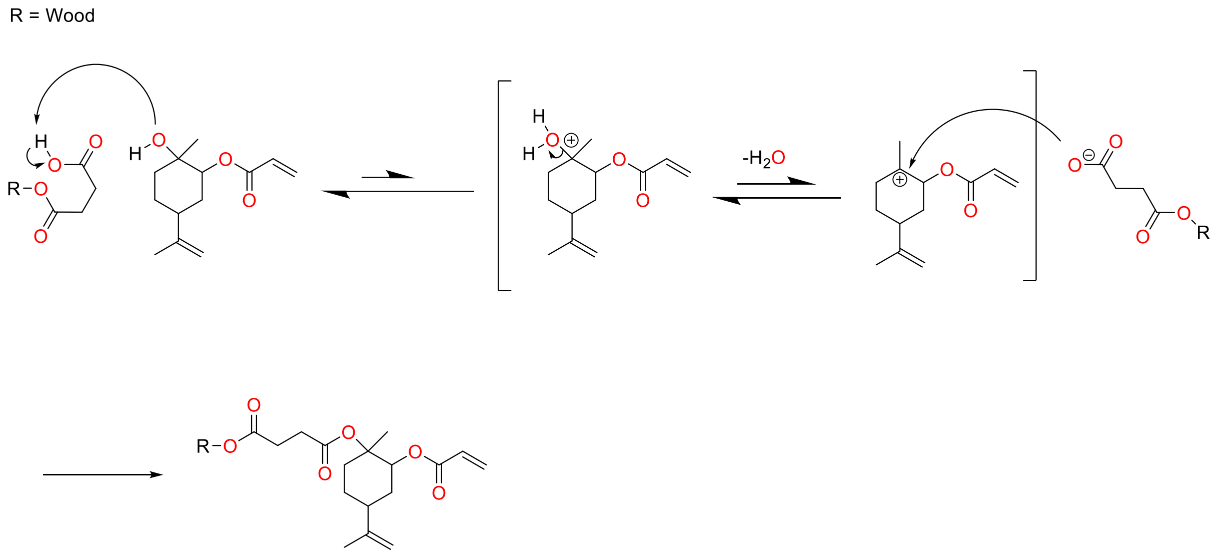

Scheme S2. Ester formation mechanism between DW-SA and LIMA monomer.

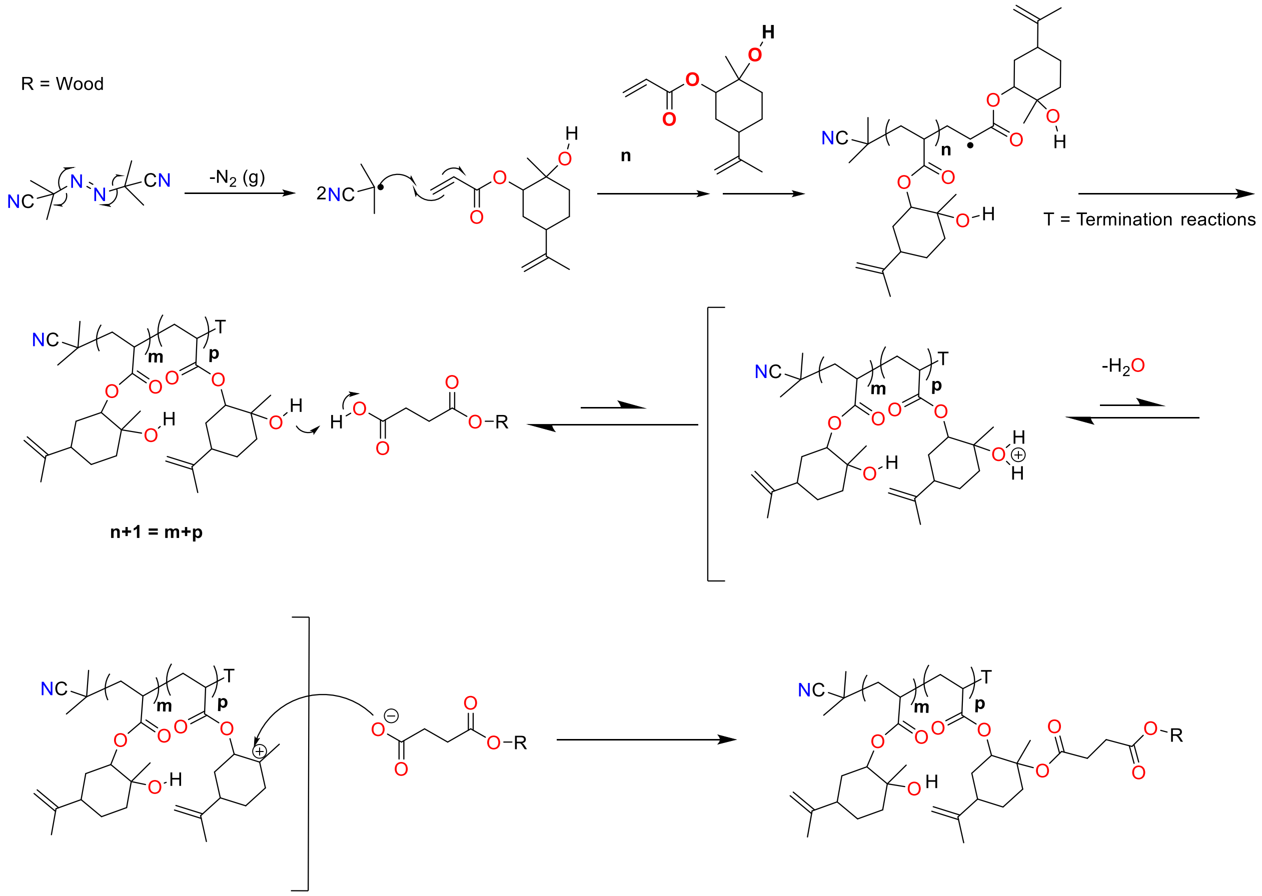

Scheme S3. Interface tailoring mechanism during the copolymerization of DW-SA and LIMA.

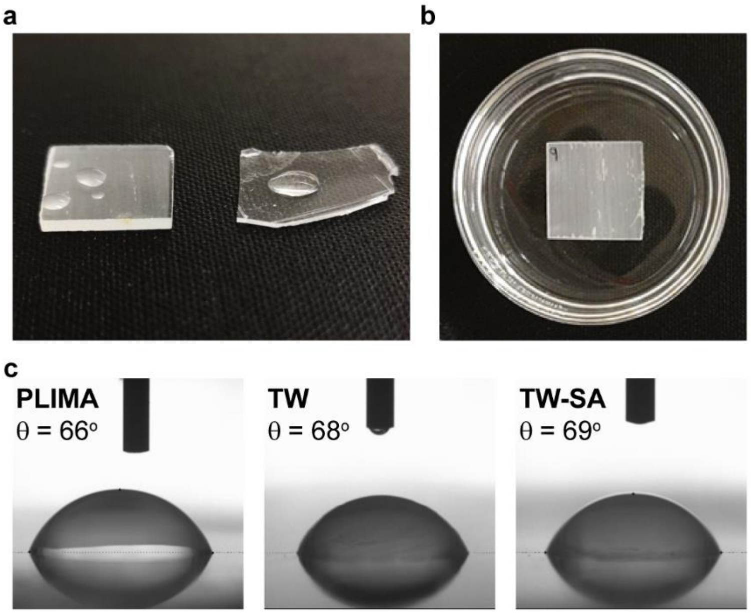

Figure S9. a) Photograph of the biobased PLIMA and transparent wood with water droplets on the surface. b) Photograph of the biobased transparent wood immersed in water (after 1 week). c) Static surface contact angle of neat PLIMA, bio-based TW and TW-SA measured with MiliQ water.

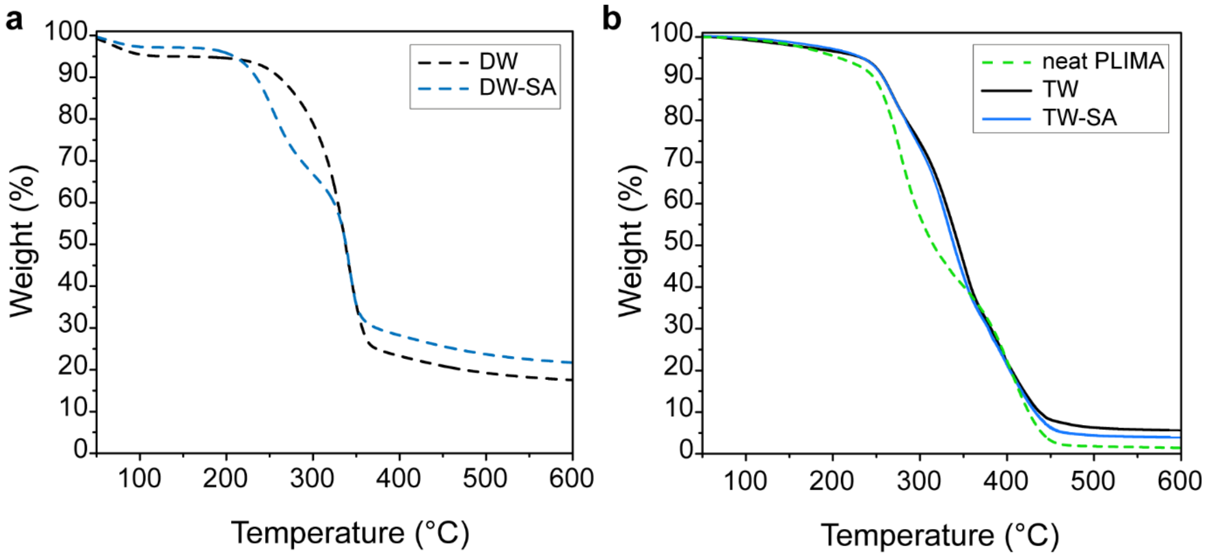

Figure S10. a) TG curves of delignified wood (DW) and succinylated substrate (DW-SA). b) TG curves of neat PLIMA, TW and TW-SA biocomposites.

Fluorinated limonene acrylate

Method.
Ring-opening acrylation of limonene oxide with 2-(trifluoromethyl)acrylic acid. The desired amount of limonene oxide (1 molar equiv.) and 2-(trifluoromethyl)acrylic acid (98%, ACROS Organics) (2 molar equiv.) was added to a 100 ml round bottom flask equipped with a magnetic stirrer. The reaction mixture was bubbled with Ar for 5 minutes at ambient temperature to remove any residual oxygen. The round bottom flask was capped and immersed in an oil bath set at 75 ˚C for 3 h. After the reaction the mixture was cooled to ambient temperature, decanted into 500 ml beaker with 200 ml deionized water and 200 ml of EtOAc. The EtOAc phase was dried with $MgSO_4$(s) and concentrated to yield the desired product fluorinated-limonene acrylate (F-LIMA) as a transparent oil (37% yield).

An increased degree of oligomerization was observed for the fluorinated F-LIMA (n = 1.85) compared to LIMA (n = 1.17). This is believed to be a consequence of the higher acidity of 2-(trifluoromethyl)acrylic acid (pKa = 2.1) compared to acrylic acid (pKa = 4.3).

Fluorinated transparent wood preparation. A mixture of F-LIMA monomer and AIBN (0.5 wt%) and was impregnated under vacuum into the DW and DW-SA substrates for 2 h. The infiltrated samples were then placed between two glass slides, packaged in aluminum foil, and polymerized at 100 °C for 48 h. The resulting fluorinated transparent wood biocomposites are termed as fluorinated TW and fluorinated TW-SA.

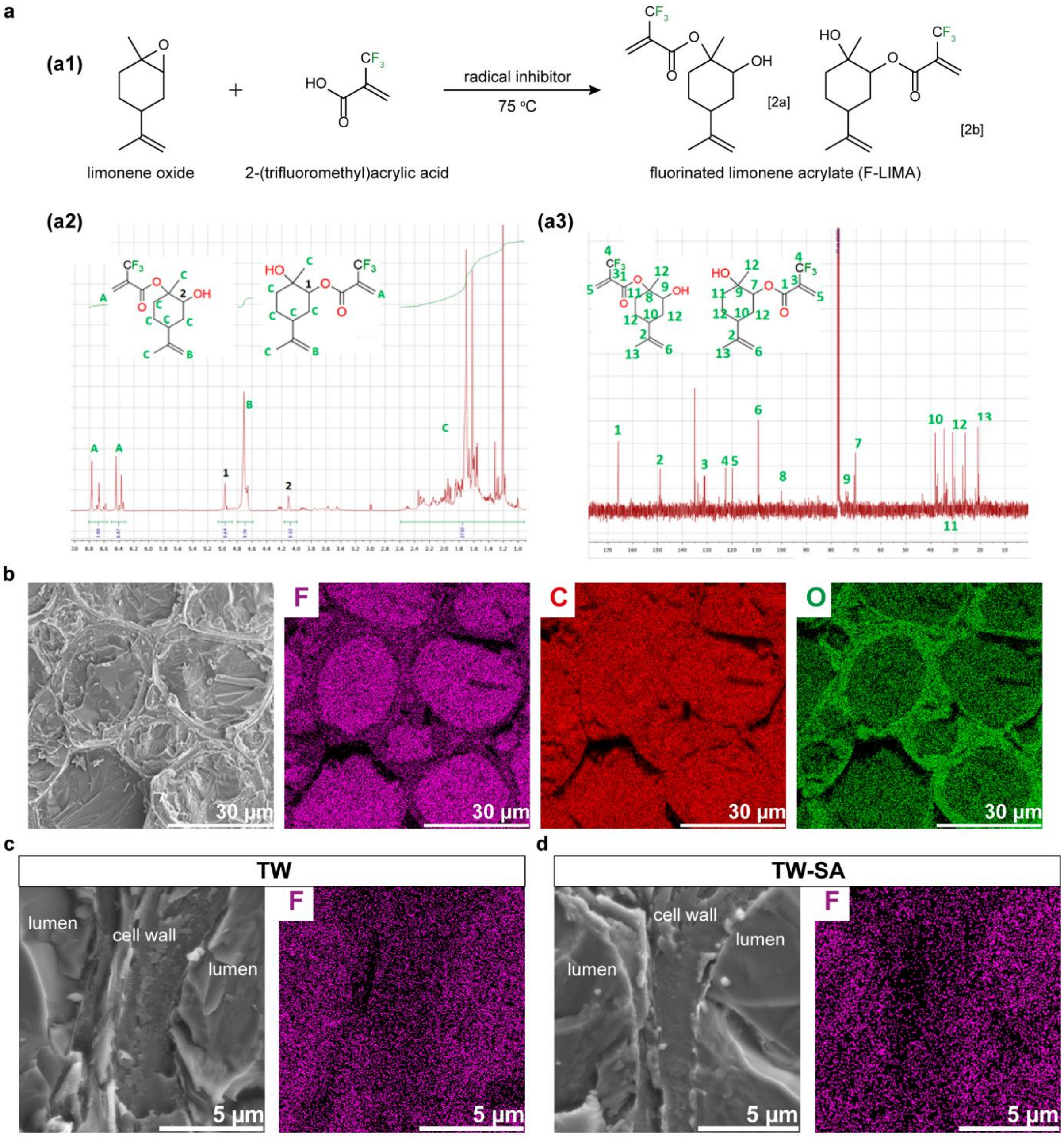

Figure S11. a) Synthesis of fluorinated limonene acrylate (F-LIMA) monomer. **(a1)** synthesis route to obtain F-LIMA: 2-hydroxy-1-methyl-4-(prop-1-en-2-yl)cyclohexyl 2-(trifluoromethyl)acrylate ([2a]) and 2-hydroxy-2-methyl-5-(prop-1-en-2-yl)cyclohexyl 2-(trifluoromethyl)acrylate ([2b]). **(a2)** ^{1}H NMR (400 MHz, Chloroform-d) δ 6.85 – 6.61 (m, 1H), 6.49 – 6.33 (m, 1H), 4.97 (d, J = 2.6 Hz, 0.64H), 4.80 – 4.60 (m, 3.7H), 4.10 (d, J = 3.5 Hz, 0.32H), 2.59 – 0.95 (m, 27H). **(a3)** ^{13}C NMR (101 MHz, Chloroform-d) δ 165.77 , 148.88 , 131.04 (d, J = 32.6 Hz), 122.52 , 119.81 , 109.39 , 100.14 , 74.12 , 70.52 (d, J = 46.6 Hz), 37.67 (d, J = 95.0 Hz), 34.06 (d, J = 80.7 Hz), 30.73 (d, J = 75.4 Hz), 28.38 – 25.13 (m), 22.37 – 18.95 (m). **b)** Cross-sectional FE-SEM and EDS maps of fluorinated TW biocomposite, where the distribution of fluorine (F), carbon (C), and oxygen (O) atoms is shown. FE-SEM and EDS map showing the distribution of fluorine atoms across the cell wall of **(c)** fluorinated TW cell wall, and **(d)** fluorinated TW-SA.

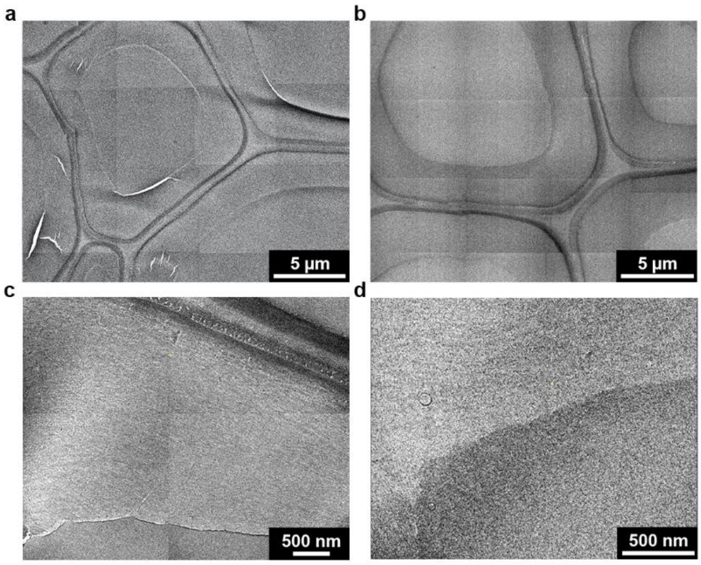

Figure S12. TEM micrographs of the wood cells of TW (a) and (c), and TW-SA (b) and (d).

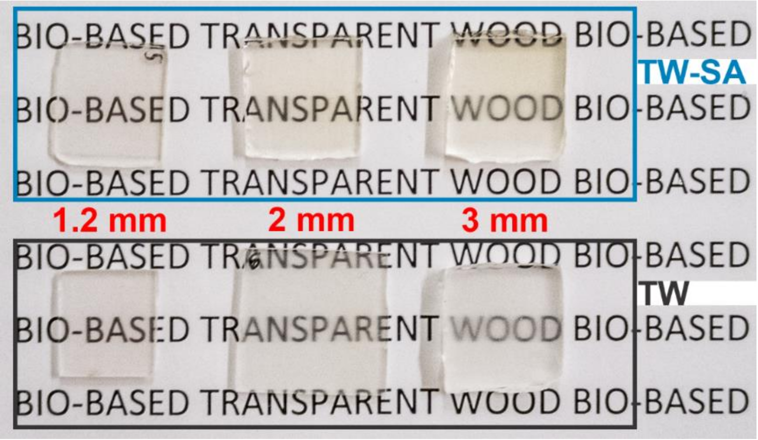

Figure S13. Photographs of TW-SA and TW composites at different thicknesses.

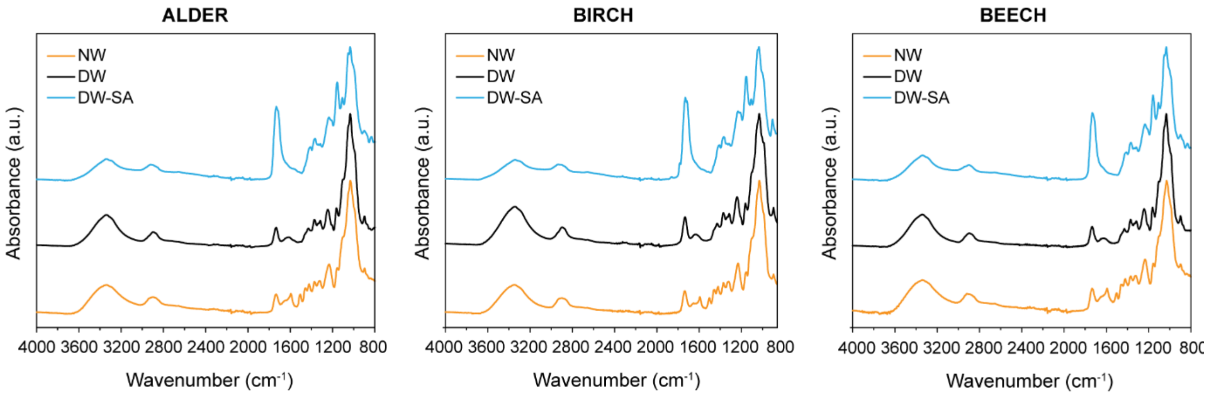

Figure S14. FTIR spectra of native wood (NW), delignified wood (DW), and succinylated (DW-SA) substrates from alder, birch and beech.

Effective axial wood cell wall modulus E_f and tensile strength σ_f

Simple estimates of axial wood cell wall properties are helpful in understanding how intrinsic cell wall properties and local stress distribution influences global mechanical properties of native wood, delignified wood and TW biocomposites. As a starting point, the concepts of relative density and cell wall volume fraction needs to be defined for wood. The volume fraction of wood cell wall V_f for native wood is

$$V_f = \rho*/\rho_f$$

Relative density is $\rho*/\rho_f$, where $\rho*$ is the density of the porous cellular material (wood) and the present notation ρ_f refers to wood cell wall density, assumed to be 1500 kg/m^3.

A simple mechanics analysis based on an idealized wood structure, inspired by Gibson and Ashby,[5] can then be used to estimate effective cell wall modulus E_f and strength σ_f for axial tensile loading. We assume that wood, as a first approximation, can be represented by a honeycomb structure according to the Schematic S1 below.

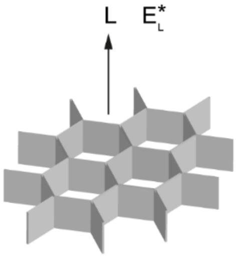

Schematic S1. A honeycomb structure loaded in tension in the longitudinal direction, carrying loads on the faces normal to longitudinal direction.[5]

For neat wood, the modulus E_L* of porous wood is then given by the cell wall modulus E_f divided by the total cross-sectional area, which results in

$$E_L*/E_f = \rho*/\rho_f = V_f$$

$$E_L* = E_f V_f$$

We can then use experimental data for E_L*, estimate V_f and calculate an effective cell wall modulus E_f. For a honeycomb structure, this expression is exact, whereas the wood structure is more complex. Ray cells in radial direction, fiber ends and inhomogeneous cell wall thickness are non-ideal features, which means that estimates of E_f for native wood are conservative; the "real" E_f will be higher.

For longitudinal wood tensile strength σ_L*, the analogous expression becomes

$$\sigma_L*/\sigma_f = \rho*/\rho_f = V_f$$

where σ_f is the effective cell wall tensile strength for loading in the longitudinal direction.

For the polymer matrix composites, we assume that the empty space in the center of the honeycomb cells is filled by polymer. We can then use analogous "rule-of-mixtures" approaches,[6] and write E_{Lc} Young's modulus in longitudinal direction of TW biocomposites as

$$E_{Lc} = E_f V_f + E_{PLIMA}(1- V_f)$$

where E_{PLIMA} is the modulus of the neat PLIMA polymer. Again, "effective" E_f can be estimated from knowledge of V_f (Table 1) and experimental data for E_{Lc} and E_{PLIMA}.

In analogy we obtain for TW biocomposite tensile strength σ_{Lc}

$$\sigma_{Lc} = \sigma_f V_f$$

Effective cell wall tensile strength can then be readily estimated for composites based on delignified native wood TW and delignified succinylated TW-SA.

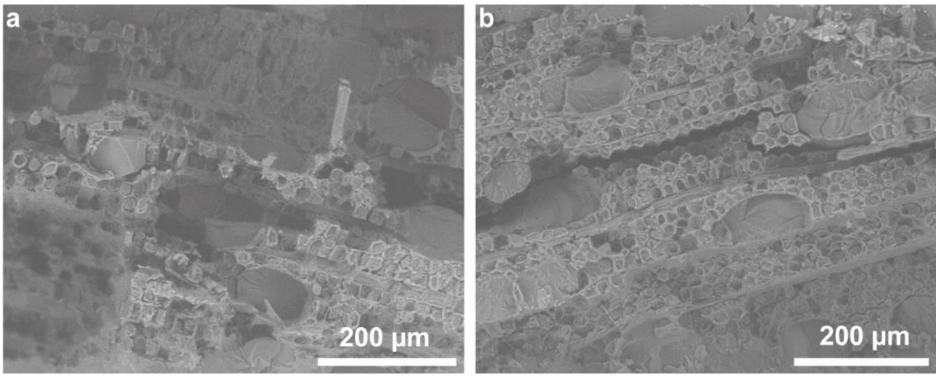

Figure S15. SEM images of the fracture surfaces of TW (a) and TW-SA (b) after loading in the longitudinal direction.

Table S2. Optical properties at 550 nm wavelength of neat PLIMA, TW and TW-SA prepared from various wood species and different wood volume fractions (V_f).

		V_f [%]	Thickness [mm]	Transmittance [%]	Haze [%]
neat PLIMA		0	1.2	95.1	2.49
Balsa	TW	6	1.2	89.8	38.7
	TW-SA	6	1.2	90.2	30.0
	TW	12	0.7	92.3[a)]	
			1.2	87.2	45.5
			2.0	79.5	62.4
			3.0	70.7	64.6
	TW-SA	12	0.7	95.1[a)]	
			1.2	88.6	41.0
			2.0	87.0	44.7
			3.0	81.4	51.3
Alder	TW	22	0.7	89.5	50.7
	TW-SA	22	0.7	89.6	42.9
Birch	TW	26	0.7	88.4	49.4
	TW-SA	26	0.7	91.6	44.3
Beech	TW	29	0.7	89.3	63.0
	TW-SA	29	0.7	88.6	61.0

[a)]Predicted value.

Prediction of transmittance for balsa-based biocomposites

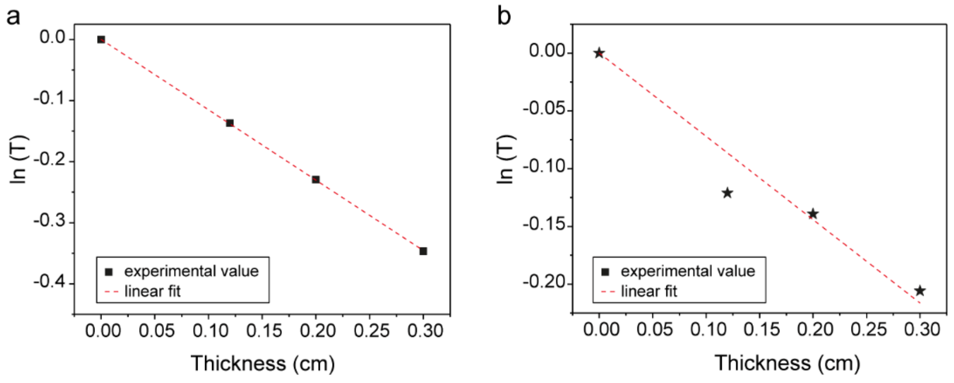

Figure S16. Fitting of total transmittance versus the sample thickness of (a) TW and (b) TW-SA prepared from balsa. The transmittance was collected at wavelength of 550 nm. The symbols represent the experimental value.

The total transmittance (T) of balsa biocomposites at a specific thickness can be predicted based on a model described by Chen et al.[7] The total transmittance can be described as

$$T = \exp(-\alpha d)$$

where α is the attenuation coefficient of the biocomposite, and d the thickness of the biocomposite.

The attenuation coefficient α is determined by fitting experimental transmittance data for TW and TW-SA at thickness of 1.2 mm, 2.0 mm and 3.0 mm. The total transmittance is assumed 100% for thickness equals to 0 mm.

The fitting in Figure S16 shows the relationship between transmittance at 550 nm wavelength and thickness of the balsa-based TW and TW-SA biocomposites. The extracted attenuation coefficients were 1.152 cm^{-1} for TW and 0.721 cm^{-1} for TW-SA.

The transmittance can then be predicted for specific thicknesses. The predicted transmittance for 0.7 mm thickness is 92.3% for TW and 95.1% for TW-SA.

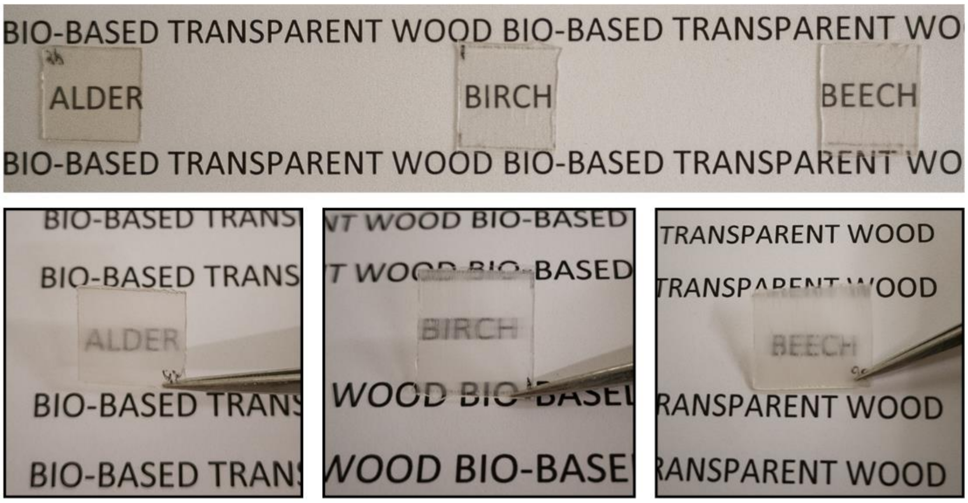

Figure S17. Photographs of TW-SA biocomposites based on alder, birch, and beech species. The photographs at the bottom demonstrate the low haze of TW-SA biocomposites as the letters behind the biocomposites appear when the samples are held above the text.

References

[1] R.-J. Zhou, T. Burkhart, *J. Appl. Polym. Sci.* **2010**, *115*, 1866.

[2] X. Wu, X. Wang, Z. Qin, W. Zhang, *J. Mater. Sci.* **2021**, *56*, 428.

[3] Y. Yao, J. Tao, J. Zou, B. Zhang, T. Li, J. Dai, M. Zhu, S. Wang, K. K. Fu, D. Henderson, E. Hitz, J. Peng, L. Hu, *Energy Environ. Sci.* **2016**, *9*, 2278.

[4] C. Montanari, P. Olsén, L. A. Berglund, *Green Chem.* **2020**, *22*, 8012.

[5] L. J. Gibson, M. F. Ashby, *Cellular Solids*, Cambridge University Press, Cambridge, **1997**.

[6] D. Hull, T. W. Clyne, *An Introduction to Composite Materials*, Cambridge University Press, Cambridge, **1996**.

[7] H. Chen, A. Baitenov, Y. Li, E. Vasileva, S. Popov, I. Sychugov, M. Yan, L. Berglund, *ACS Appl. Mater. Interfaces* **2019**, *11*, 35451.
